# Prognostic value and immune landscapes of cuproptosis-related lncRNAs in esophageal squamous cell carcinoma

**DOI:** 10.18632/aging.205089

**Published:** 2023-10-06

**Authors:** Xiang Zhang, Nan Feng, Bo Wu, Zishun Guo, Tiewen Pan, Xiandong Tao, Hongyang Zheng, Wenxiong Zhang

**Affiliations:** 1 Department of Thoracic Surgery, The Second Affiliated Hospital of Nanchang University, Nanchang 330006, China; 2 Department of Thoracic Surgery, Third Affiliated Hospital of Naval Medical University, Shanghai 201805, China

**Keywords:** cuproptosis, lncRNAs, esophageal squamous carcinoma, prognosis, drug sensitivity

## Abstract

Background: Precisely forecasting the prognosis of esophageal squamous cell carcinoma (ESCC) patients is a formidable challenge. Cuproptosis has been implicated in ESCC pathogenesis; however, the prognostic value of cuproptosis-associated long noncoding RNAs (CuRLs) in ESCC is unclear.

Methods: Transcriptomic and clinical data related to ESCC were sourced from The Cancer Genome Atlas (TCGA). Using coexpression and Cox regression analysis to identify prognostically significant CuRLs, a prognostic signature was created. Nomogram models were established by incorporating the risk score and clinical characteristics. Tumor Immune Dysfunction and Rejection (TIDE) scores were derived by conducting an immune landscape analysis and evaluating the tumor mutational burden (TMB). Drug sensitivity analysis was performed to explore the underlying molecular mechanisms and guide clinical dosing.

Results: Our risk score based on 5 CuRLs accurately predicted poorer prognosis in high-risk ESCC patients across almost all subgroups. The nomogram that included the risk score provided more precise prognostic predictions. Immune pathways, such as the B-cell receptor signaling pathway, were enriched in the datasets from high-risk patients. High TMB in high-risk patients indicated a relatively poor prognosis. High-risk patients with lower TIDE scores were found to benefit more from immunotherapy. High-risk patients exhibited greater responsiveness to Nilotinib, BI-2536, P22077, Zoledronate, and Fulvestrant, as revealed by drug sensitivity analysis. Real-time PCR validation demonstrated significant differential expression of four CuRLs between ESCC and normal cell lines.

Conclusions: The above risk score and nomogram can accurately predict prognosis in ESCC patients and provide guidance for chemotherapy and immunotherapy.

## INTRODUCTION

Esophageal cancer (ESCA) is a common and fatal malignancy that affects people worldwide [1]. The main histologic subtype of ESCA is esophageal squamous cell carcinoma (ESCC) [2]. Unfortunately, the lack of early clinical signs and symptoms of Delayed diagnosis of ESCC leads to untreatable 75% of patients [3]. In addition, current clinical assessment metrics rely primarily on TNM staging [4]. Inaccurate prognostic prediction of ESCC patients has been reported. Patients with early ESCC can be treated by surgery; however, most ESCC patients are diagnosed in advanced stages, and there are few drug options for patients with advanced ESCC, with chemotherapeutic agents that are more cytotoxic to patients and have worse side effects. Recently, there have been breakthroughs in the treatment of ESCC patients with Immune Checkpoint Inhibitors (ICIs) targeting programmed cell death protein 1 (PD1), programmed cell death 1 ligand 1 (PDL1), or cytotoxic T lymphocyte antigen 4 (anti-CTLA4) [5, 6]. However, only a small percentage of ESCC patients benefit from immunotherapy, while others develop innate resistance. Therefore, there is an urgent need for biomarkers that can help predict prognosis and guide therapy in ESCC patients at this stage.

**Figure 1:**
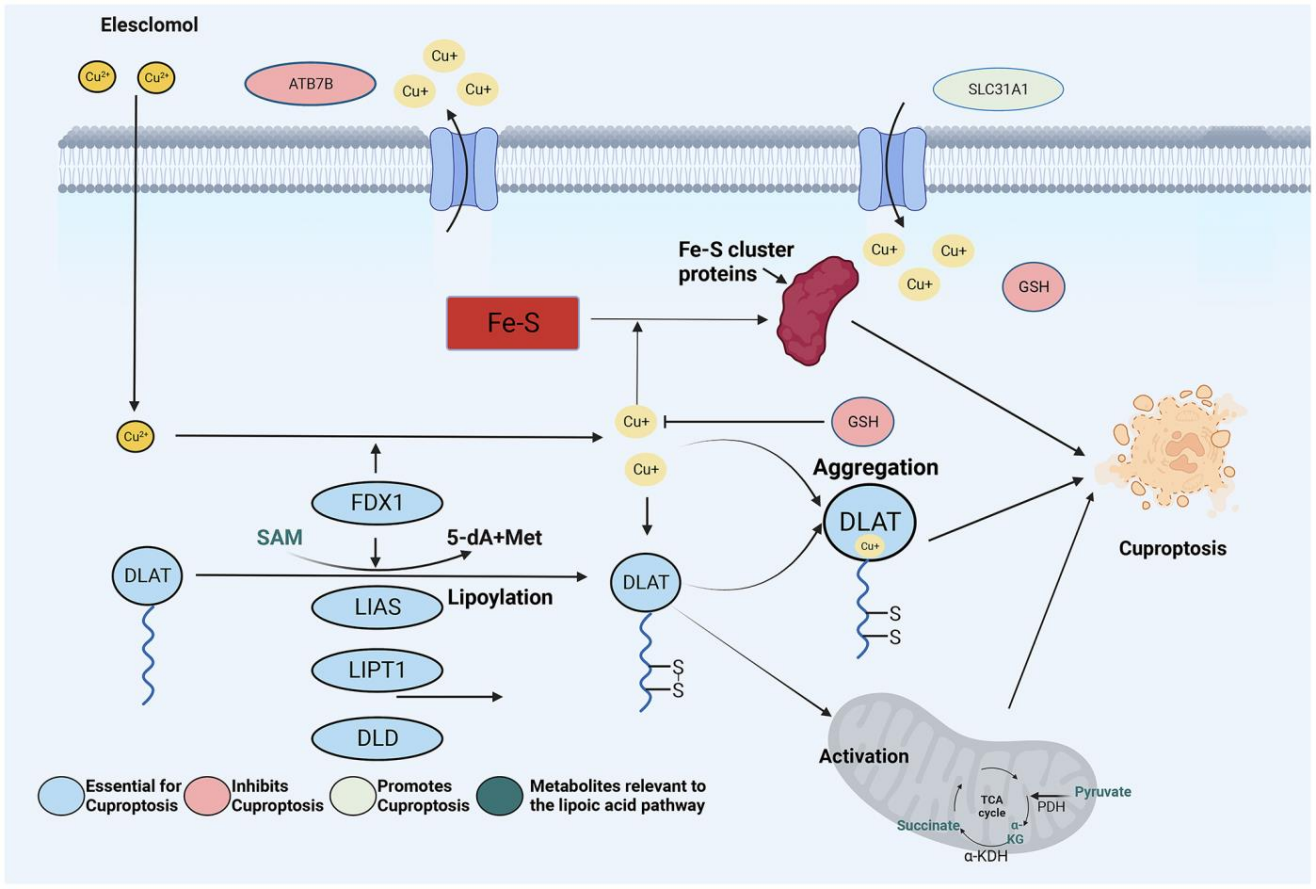
Schematic illustration indicates the mechanism of cuproptosis induction and research design.

Numerous studies have investigated various forms of regulated cell death that shape the biological and therapeutic response to ESCC, including ferroptosis and macrophages. Excitingly, a recent study by Tsvetkov and colleagues published in the journal Science confirmed that copper-induced regulated cell death, also known as cuproptosis, is a newly discovered form of regulated cell death that is distinct from apoptosis, sepsis, and iron death [7]. Cuproptosis is intricately linked to the mitochondria, and the underlying mechanism entails the direct interaction of copper with lipidized constituents in the tricarboxylic acid cycle (Figure 1) [8]. As a result of this interaction, fatty acylated protein aggregation occurs, along with iron-sulfur cluster protein loss. This cause stress and protein toxicity, which eventually results in cell death.

Prognostic models of cuproptosis-related long noncoding RNAs (CuRLs) for head and neck squamous cell carcinoma and gastric cancer have already been established [9, 10]. However, many CuRLs remain underexplored. Therefore, we aimed to analyze the immunological association of CuRLs with ESCC and construct a prognostic model using the expression of lncRNAs from ESCC patients in the TCGA database. We examined the association between the prognostic models developed and the immune infiltration of tumors, response to immunotherapy, and susceptibility to targeted medications. The results of this study will facilitate prognostication and immunotherapeutic approaches for ESCC, as well as personalized patient management.

## MATERIALS AND METHODS

### Data extraction and collation and CuRL screening

The transcriptome data of patients with esophageal cancer were obtained from the TCGA-GDC website, followed by annotation of the ESCC dataset. R language was used to preprocess the commercial data to acquire clinically informative information on the pathology of the clinical specimens. Simultaneously, protein-coding genes and lncRNAs were converted using R language, and lncRNAs were extracted from transcriptome analysis set. After a series of exclusions, a cohort of 94 ESCC patients was analyzed for follow-up purposes. After merging the lncRNAs with the clinical information, the patients were divided 1 to 1 between a test (*n* = 47) group and a training (*n* = 47) group. The GSE53625 cohort (containing 179 ESCC patients) was downloaded from the Gene Expression Omnibus (GEO) for external validation of the predictive power of the prognostic model.

### Identification of CuRLs

The expression of 25 cuproptosis-related genes (ATOX1, ATP7A, ATP7B, CCS, COX7B, CP, DLAT, DLD, DLST, FDX1, GCSH, LIAS, LIPT1, LIPT2, MITD1, NDUFA1, NDUFA2, NDUFB1, NDUFB2, PDHX, PIH1D2, SLC22A5, SLC23A2, SLC31A1, SLC6A3) was obtained by previous studies. To identify CuRLs clearly associated with ESCC (|Pearson R| > 0.4, *p* < 0.001), Pearson-related analysis was performed [11], CuRLs were screened for prognosis-related lncRNAs (*p* < 0.05) using one-way Cox regression analysis [12], and a forest plot was drawn.

### Prognostic risk score

The LASSO Cox regression algorithm was used to calculate the lncRNAs with the best prognostic value, which were used to create a risk score [13]. In the next step, we performed multivariate Cox regression calculations with the obtained optimal lncRNAs to model the associated risk score of prognosis, and the following equation for the ESCC case risk score was obtained: risk score = (AC021321.1 × −2.51860599311819) + (LINC01775 × −0.850960975441784) + (LINC00601 × 0.671071886012604) + (EWSAT1 × 0.616117492510334) + (AC138696.2 × −0.807041898484958). According to the median risk score obtained from the prognostic signature, the patients were divided into two groups: high risk and low risk. Then, Kaplan-Meier (KM) curves were generated separately for the training and test groups to determine whether there was a difference in overall survival [14]. To determine the degree of correlation between the model and patient clinical characteristics, we utilized two highly effective analytical tools: receiver operating characteristic (ROC) curves and area under the curve (AUC) calculations [15]. Decision curve analysis (DCA) was utilized to demonstrate the clinical applicability of this model. By employing these methods, we were able to generate comprehensive and insightful results. Principal component analysis (PCA) was used to visualize the spatial distribution of high-risk and low-risk samples in esophageal tumor cases [16].

### Nomogram plots and calibration plots

Nomogram plots were constructed by combining the risk score and various clinical data in R language software to analyze the predicted 1-, 2-, and 3-year survival rates of ESCC patients, and we employed calibration curves. These curves provided us with valuable insights into the accuracy of our predictions, allowing us to make any necessary adjustments and improvements to the model [17].

### Analysis of tumor mutation burden (TMB) and gene set enrichment analysis (GSEA)

Using GSEA 4.3.2 software, enriched pathway analysis was conducted in both high- and low-risk groups using five distinct methods: Pathway Interaction Database (PID), Gene Ontology (GO), REACTOME, Wiki Pathways (WP), and Kyoto Encyclopedia of Genes and Genomes (KEGG) [18, 19]. |NES| > 1 and FDR < 0.25 were used as criteria. We utilized the R package “maftools” to compare the association between risk score and TMB [20]. KM curves were used to compare the overall survival (OS) of the high and low TMB groups.

### Tumor microenvironment characteristics, drug sensitivity and mutation data

We used several algorithms, including XCELL, TIMER, QUANTISEQ, MCPcounter, EPIC, CIBERSORT and CIBERSORT-abs, to evaluate the association between tumor immune infiltration and high/low-risk groups [21]. To assess drug sensitivity among different risk groups, the “prognostic” package in R was used [22], which predicts the 50% inhibitory concentration (IC50) of commonly administered chemotherapeutic and immunological agents for ESCC.

### Cell culture and qRT-PCR

Human specimens were collected from patients who underwent ESCC resection at the Department of Thoracic Surgery of the Second Affiliated Hospital of Nanchang University. A total of 6 pairs of ESCC specimens and paracancerous specimens were collected. After separation of the samples, some of the esophageal cancer tissues were rapidly frozen in liquid nitrogen and then stored in a −4°C refrigerator to avoid degradation. Normal human esophageal epithelial cell lines (HEEC) from JiNiu Biologicals (China) and TE-1, KYSE-30, KYSE-410, and KYSE-520 cell lines from Wuhan Procell (China) were maintained in DMEM (Gibco, USA) (HyClone, USA) replenished in 10% FBS at 37°C and 95% air with 5% CO2.

RNA was extracted from the cells using TRIzol (Invitrogen, USA) and RNA extraction kits. RNA was converted into cDNA using the PrimeScript RT Reagent Kit (Takara, Japan) and analyzed for gene expression via qRT-PCR. Primers are shown in Supplementary Table 1.

### Statistical analysis

**Figure 2:**
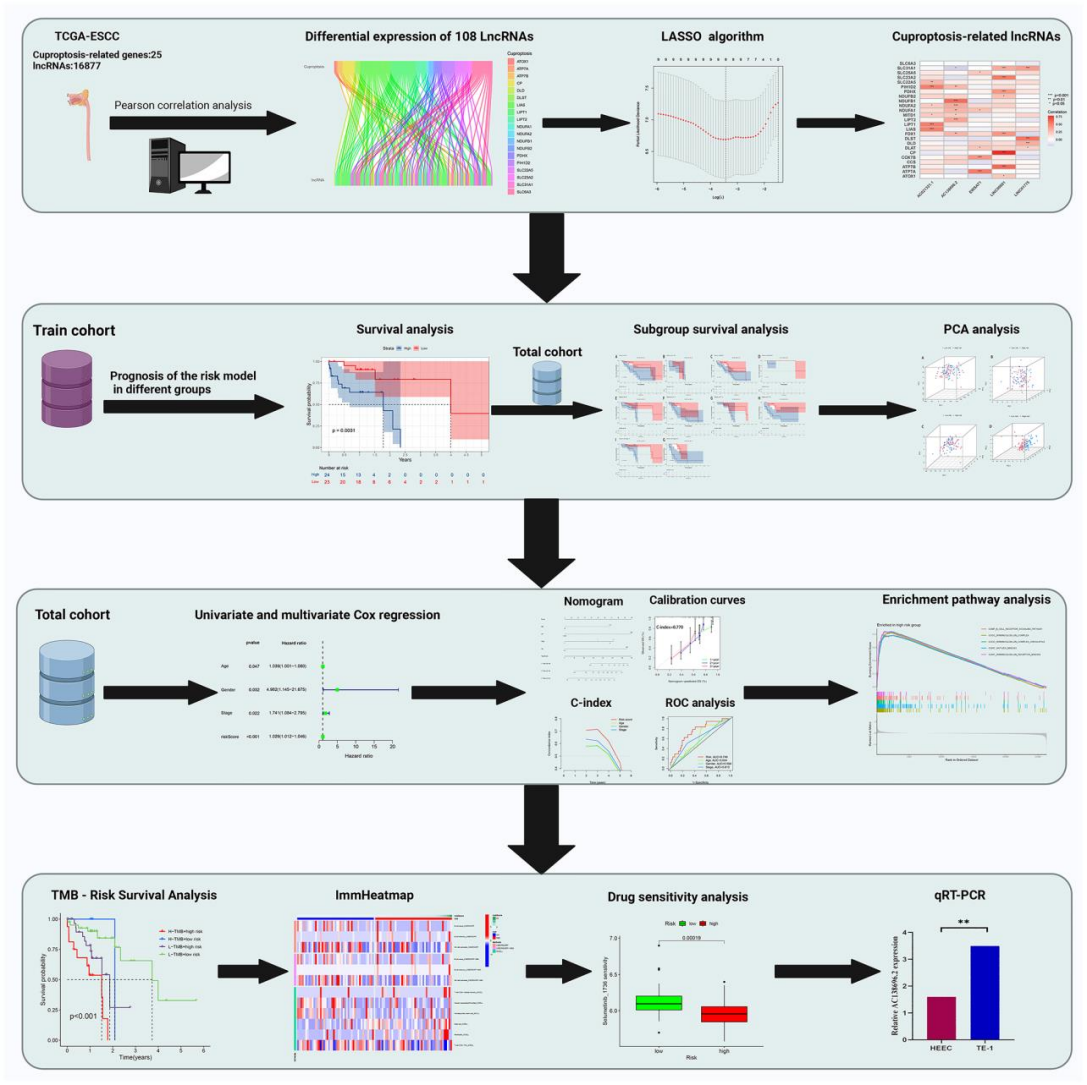
Overall flow diagram of the study.

Data were statistically divided and visualized by R language software, and data processing mainly employed the Perl programming language. Statistical differences between the groups were calculated using Student’s *t*-test and analysis of variance (ANOVA). OS in the two groups was compared using KM analysis. Univariate, LASSO, and multifactorial Cox regression analyses were utilized to assess prognostic significance. Gene expression correlations were obtained using Pearson correlation analysis. Prognostic feature reliability and sensitivity were estimated using ROC curves and AUCs. Statistically significant differences were defined as bilateral *p* < 0.05 (Figure 2).

### Availability of data and material

The data sets used and/or analyzed during the current study are available from the corresponding author on reasonable request.

## RESULTS

### Screening of lncRNAs associated with cuproptosis and the prognosis of esophageal cancer

**Figure 3:**
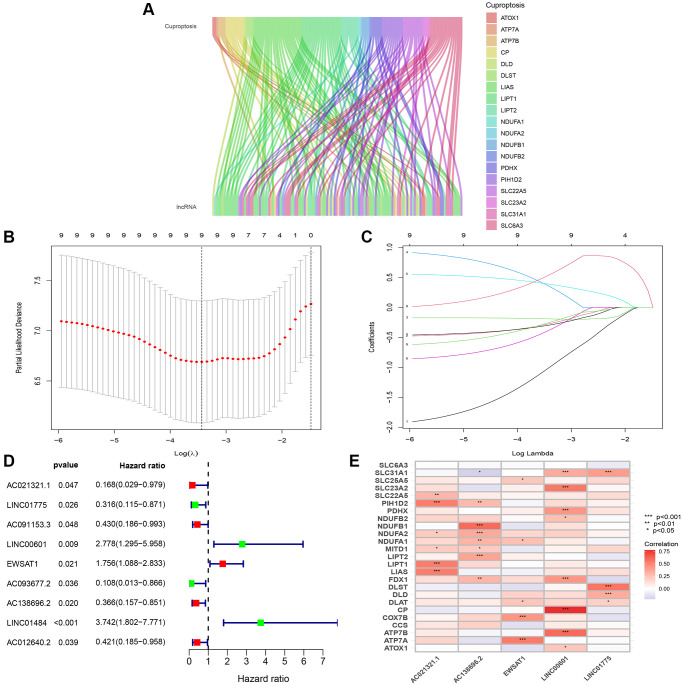
**Identification of significant prognosis CuRLs.** (**A**) Sankey diagram depicting the relationships between 25 cuproptosis-related genes and CuRLs co-expression. (**B**) LASSO Cox regression analysis revealed 9 CuRLs based LASSO cross validation plot. (**C**) LASSO coefficient of 9 CuRLs. (**D**) Forest plot of 9 one-way Cox regressions derived with prognosis-related lncRNAs. (**E**) Correlation heatmap of the association of 5 signature lncRNAs with cuproptosis-related genes.

In the first step of the study, the ESCC dataset was retrieved from the TCGA-GDC website, and a total of 16877 lncRNAs were screened. Subsequently, 25 cuproptosis-related genes were correlated with lncRNAs using Pearson correlation analysis. A total of 108 lncRNAs were identified in this step (Figure 3A). Next, the data of 94 ESCC patients were obtained after excluding three patients who did not have tumor or survival data. The comprehensive clinicopathological data of the patients are detailed in Table 1. Subsequently, the 94 patients with ESCC were randomly divided equally into training and test groups. The prognostic risk score was built using the training group, and the test group was used for validation. Nine prognostic CuRLs were identified via one-way Cox analysis in the training group (Figure 3D). LASSO regression analysis was subsequently carried out to identify lncRNAs that are correlated with the prognosis of ESCC, and 5 CuRLs (AC021321.1, LINC01775, LINC00601, EWSAT1, and AC138696.2) were identified (Figure 3B, 3C). The LASSO regression analysis results are shown in Supplementary Table 2. The corrplot showed a strong association between these 5 CuRLs and cuproptosis-related genes (Figure 3E).

**Table 1:** Demographic and baseline characteristics of ESCC cases from the TCGA.

**Characteristics**	**Entire cohort (*n* = 94)**	
	***n***	**%**
**Gender**		
Female	14	15.79
Male	80	74.21
**Age**		
<65	71	74.74
≥65	23	25.26
**Stage**		
Stage I	7	7.37
Stage II	55	58.95
Stage III	26	27.34
Stage IV	4	4.21
Unknown	2	2.13
**T stage**		
T0	8	8.42
T1	32	33.68
T2	48	51.56
T3	4	4.21
T4	2	2.13
Unknown	8	8.42
**N stage**		
N0	54	57.89
N1	28	29.47
N2	6	6.32
N3	3	3.16
Unknown	3	3.16
**M stage**		
M0	82	87.37
M1	4	4.21
Unknown	8	8.42

Abbreviations: ESCC: Esophageal squamous cell carcinoma; M: metastasis; N: lymph node; T: Tumor.

### Prognostic modeling and validation of CuRLs

**Figure 4:**
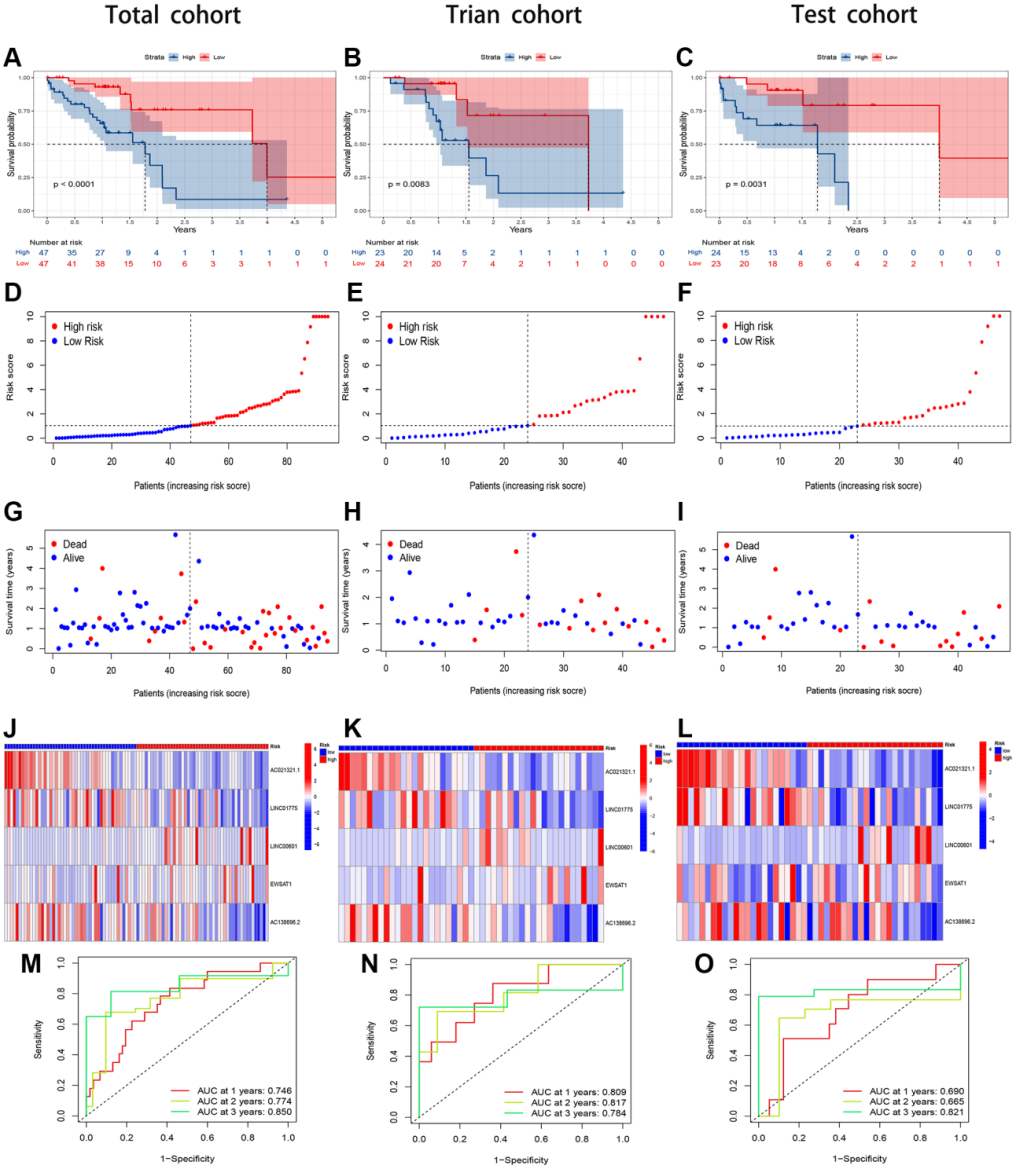
**Evaluation of the CuRLs prognostic signature in the total, training and test cohorts.** (**A**–**C**) Overall survival Kaplan-Meier survival curves. (**D**–**F**) Overall survival risk score. (**G**–**I**) Distribution of survival time and survival status. (**J**–**L**) Heatmap of 5 lncRNA expressions. (**M**–**O**) 1-, 2-, and 3-years overall survival area under the ROC curve of the signature.

The regression coefficients of the 5 lncRNAs were obtained by multifactorial Cox regression of the training group in the previous step, and each patient’s risk score was calculated. Based on the median risk score, the patients were divided into two groups: a low-risk group and a high-risk group. We performed KM survival analysis for both groups and obtained survival curves showing that the high-risk group had a significantly lower survival rate than the low-risk group (Figure 4B). The feasibility of the risk score was verified using the ROC curve, with AUC values of 0.809, 0.817, and 0.784 for 1, 2, and 3 years, respectively (Figure 4N). The scatter plots and heatmaps for the training set demonstrated that the low-risk group had significantly longer survival times than the high-risk group in the risk assessment (Figure 4E, 4H, 4K). We used the same methodology for the total and test groups and performed KM survival analysis. The results showed that the survival rate was significantly better in the low-risk group than in the high-risk group (Figure 4A, 4C). Scatter plots and heatmaps were used to assess the risk in both groups and revealed a significant difference in survival time between the low-risk and high-risk groups (Figure 4D, 4G, 4J, 4F, 4I, 4L). The AUC values for the ROC curves were 0.746, 0.774, and 0.850 at 1, 2, and 3 years, respectively, for the total cohort and 0.690, 0.665, and 0.821 for the test group (Figure 4M, 4O). The CuRL risk assessment model showed superior prognostic ability in all groups.

### Subgroup analysis and principal component analysis

Patients were stratified into different groups and subsequently analyzed for survival using the KM method, and we found that the risk assessment model could make meaningful predictions for most classifications except for the ≥65 years subgroup and the female subgroup (Supplementary Figures 1B and 2B). In all other subgroups, the overall survival was significantly higher in the low-risk group than in the high-risk group (Supplementary Figures 1A, 1C–1F and 2A, 2C, 2D), with a statistical significance of *p* < 0.05. The inability to accurately predict the prognosis in the age ≥65 years and female subgroups may be explained by the small sample sizes in the TCGA-GDC database, which prevented accurate analysis. PCA compared the expression spectra of the high- and low-risk groups, including the total spectrum of expression, cuproptosis gene expression profiles, expression profiles of lncRNAs associated with 25 cuproptosis genes, and CuRL expression profiles (Supplementary Figure 3A–3D). Our analysis indicated that the developed prognostic model could effectively distinguish between the two risk groups.

### Clinical value validation of the model for risk prediction

**Figure 5:**
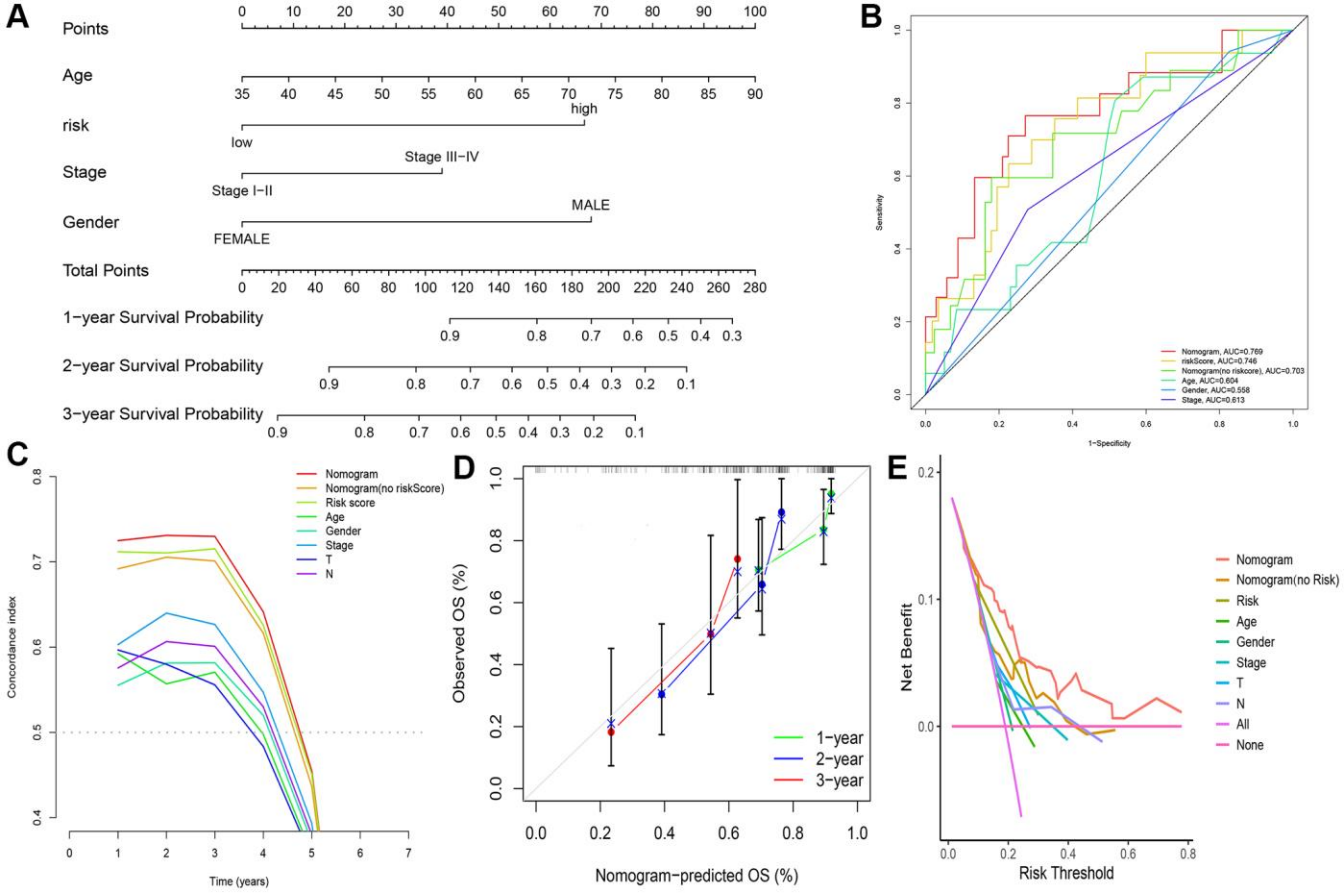
**Construction of nomogram and validation of its predictive ability.** (**A**) Nomogram to predict the overall survival of ESCC. (**B**) ROC curves for the risk score and other clinical characteristics. (**C**) C-Index curve for the risk score and other clinical characteristics. (**D**) Calibration curves for 1, 2, and 3 years of nomogram. (**E**) DCA curve of the nomogram.

To validate the clinical value of the risk assessment model, we integrated the clinicopathological characteristics of 94 ESCC patients, including age, gender, stage, and risk score. Subsequently, univariate and multivariate Cox regression analyses were conducted for both the risk score and clinicopathological characteristics, as presented in Table 2. The risk score had remarkable independent prognostic value in both the univariate and multivariate Cox regression analyses (*p* < 0.05) (Supplementary Figure 4A, 4B). We used risk assessment and other clinicopathological characteristics to construct nomograms to predict 1-, 2-, and 3-year prognosis (Figure 5A and Supplementary Figure 4C). Calibration curves were used to validate the nomograms (Figure 5D). DCA demonstrated the clinical applicability value of this model (Figure 5E). The nomogram’s ability to predict survival was evaluated by assessing the concordance between predicted and actual survival using the C-index, which was 0.770 for the nomogram containing the risk score. We also constructed a nomogram model without the risk score and validated it using calibration curves, which had a C-index of 0.686. This demonstrated that the prediction model containing the risk score outperformed the conventional prediction model. The nomogram marker exhibited a higher C-index than any other risk factor (Figure 5C). The nomogram model ROC curve had an AUC value of 0.769 for the risk score, surpassing the AUC values of other pathological features, thereby indicating strong predictive ability when compared to other clinicopathological features (Figure 5B).

**Table 2:** Univariate and multivariate cox regression analysis based on risk factors.

**Characteristic**	**Univariate analysis**			**Multivariate analysis**		
	**HR**	**HR (95% CI)**	***P*-value**	**HR**	**HR (95% CI)**	***P*-value**
**Age**	1.039	(1.001, 1.080)	0.047	1.042	(1.000, 1.086)	0.051
**Gender**	4.982	(1.145, 21.675)	0.032	3.801	(0.860, 16.804)	0.078
**Stage**	1.741	(1.084, 2.795)	0.022	1.338	(0.795, 2.249)	0.273
**Risk Score**	1.106	(1.041, 1.175)	<0.001	1.023	(1.005, 1.042)	0.014

Abbreviations: CI: Confidence interval; HR: Hazard ratio.

### External validation of prognostic characterization

To verify the predictive ability of the model constructed with the risk score, we validated the model using the GSE53628 cohort, and the KM survival analysis revealed that patients in the high-risk group had a worse prognosis than those in the low-risk group, with a statistically significant difference (*p* < 0.001) (Supplementary Figure 5A). The feasibility of the risk score was verified using ROC curves with AUC values of 0.682, 0.698 and 0.582 at 1, 3 and 5 years, respectively (Supplementary Figure 5B). Subsequently, we performed univariate and multivariate Cox regression analyses of the risk score and clinicopathologic characteristics, and the risk score had significant independent prognostic value in both the univariate and multivariate Cox regression analyses (*p* < 0.001) (Supplementary Figure 5C, 5D). We similarly constructed nomograms (Supplementary Figure 5E) predicting 1-, 3-, and 5-year prognosis using the risk score and other clinicopathologic characteristics (Supplementary Figure 5F). Validating the nomograms with calibration curves, we found high predictive accuracy.

### Identification of the biological pathways associated with the 5 CuRLs

**Figure 6:**
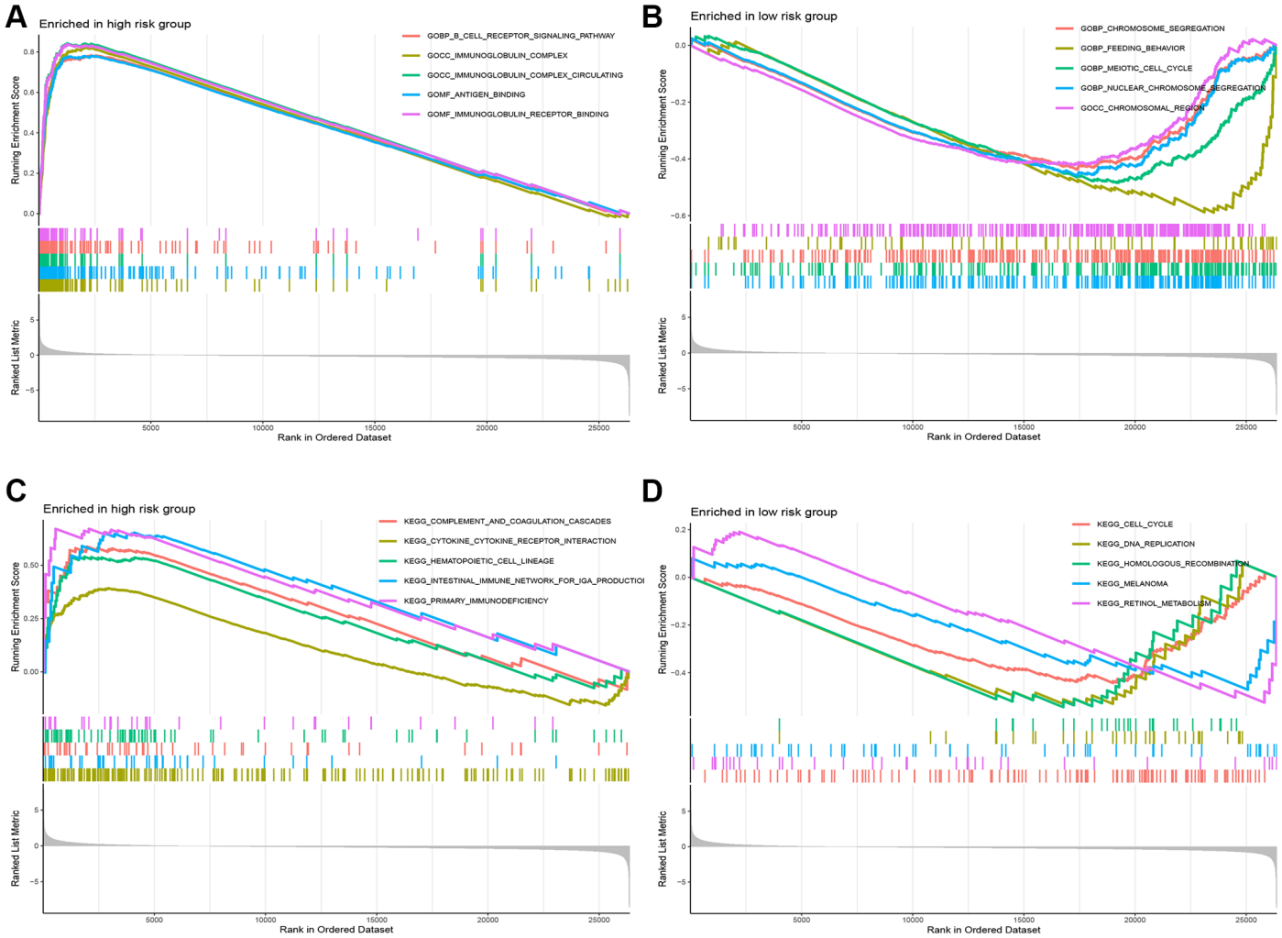
**Functional analysis between the high-risk and low-risk groups.** (**A**, **B**) The pathways of GO enriched in the low- and high-risk group. (**C**, **D**) The pathways of KEEG enriched in the low- and high-risk group.

**Figure 7:**
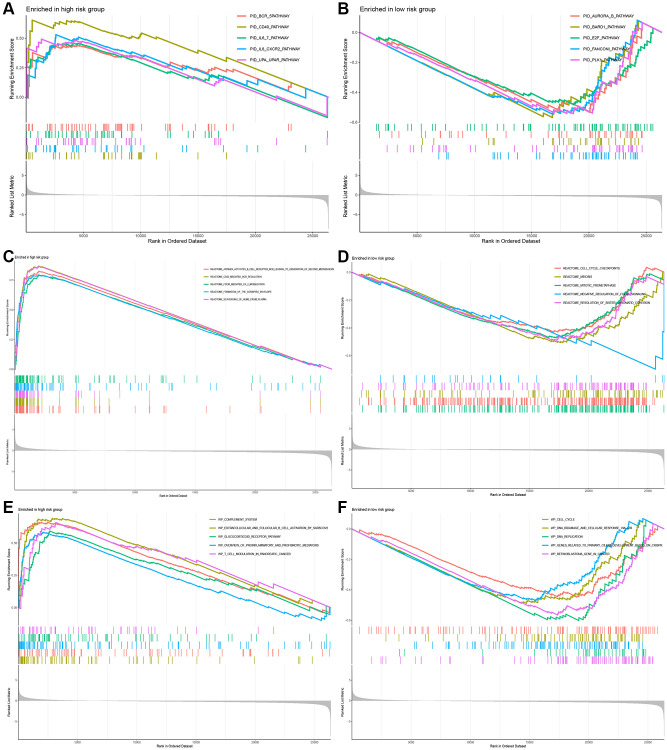
**Functional analysis between the high-risk and low-risk groups.** (**A**, **B**) The pathways of PID enriched in the low- and high-risk group. (**C**, **D**) The pathways of REACTOME enriched in the low- and high-risk group. (**E**, **F**) The pathways of WP enriched in the low- and high-risk group.

To further illustrate the association of the five CuRLs with biological processes, GSEA was performed, which yielded 332 GO functions, 28 KEGG pathways, 27 PID pathways, 153 REACTOME-related pathways and 68 WP-related pathways. GO analysis revealed that the major pathways for the lncRNAs were associated with BP terms such as cell receptor signaling, CC terms such as immunoglobulin complexes and circulating immunoglobulin complexes, and MF terms such as immunoglobulin receptor binding and antigen binding (Figure 6A, 6B). The cytokine receptor interaction pathway ranked as the top KEGG signaling pathway in our analysis, followed by hematopoietic stem cells, complement and coagulation system, primary immunodeficiency, and intestinal immune network for IGA production (Figure 6C, 6D). The top five PID signaling pathways were the CD40 pathway, IL8_CXCR2 pathway, TOLL_ENDOGENOUS pathway, BCR_5 pathway and PLK1_pathway (Figure 7A, 7B). The REACTOME pathways and top five WP signaling pathways are shown in Figure 7C–7F.

### Effect of TMB and CuRL signaling on chemotherapy

**Figure 8:**
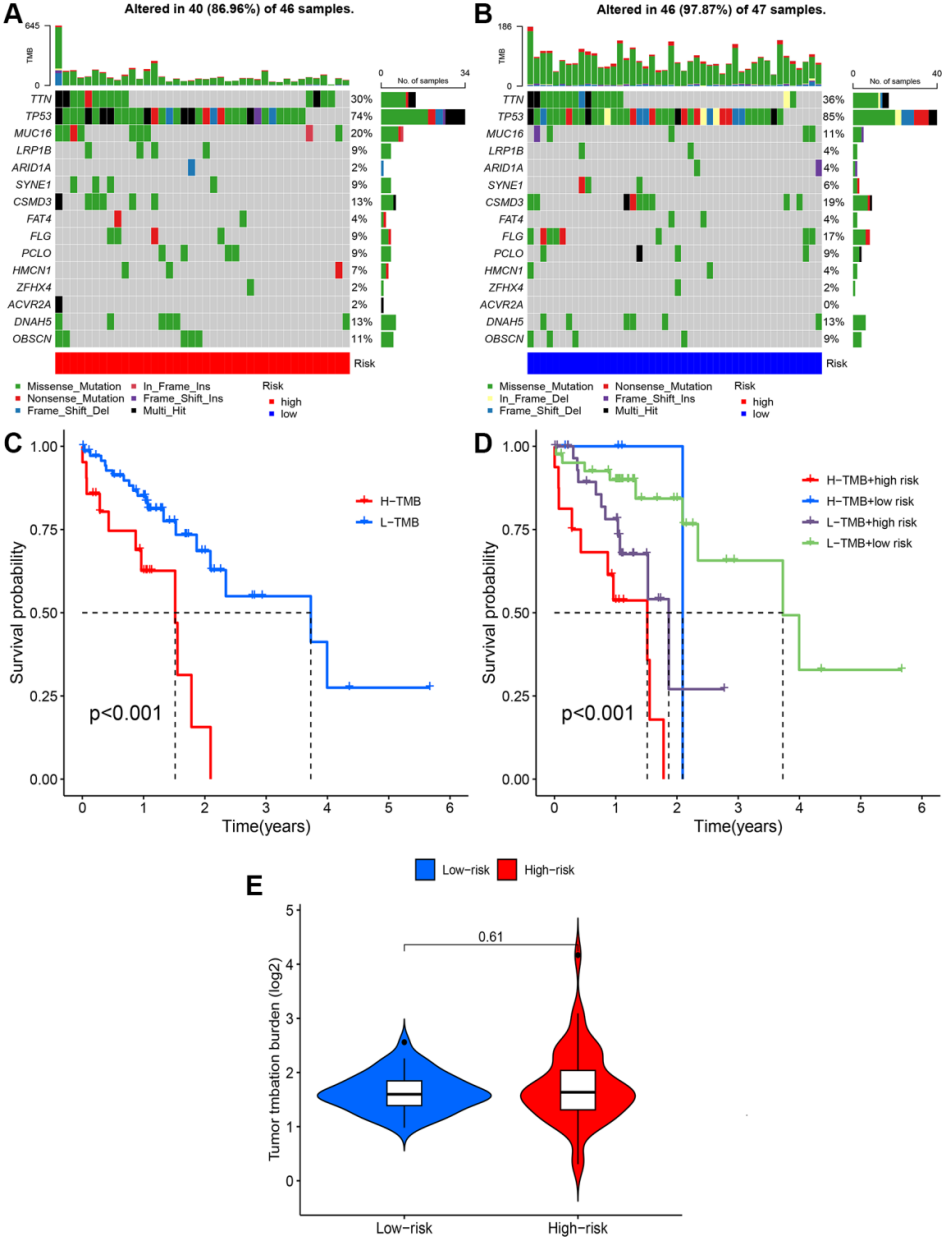
**Tumor mutation burden (TMB) analysis.** (**A**, **B**) Waterfall plots of somatic mutation characteristics in the two groups. (**C**) Kaplan-Meier survival curves between the high- and low-TMB groups. (**D**) Kaplan-Meier survival curves between the four groups. (**E**) TMB between the low-risk and high-risk groups.

The somatic mutation database was downloaded to investigate the mutation rates in the high- and low-risk groups from the TCGA database. The 10 genes with the highest mutation rates were TP53, TTN, CSMD3, MUC16, SYNE1, LRP1B, PCLO, FLG, HMCN1, and SYNE1. Among them, TP53, TTN, CSMD3, MUC16 and SYNE1 were the genes most commonly mutated in ESCC (Figure 8A, 8B). Furthermore, the high-risk group had a significantly higher TMB than the low-risk group (*p* < 0.05) (Figure 8C, 8D), and patients with high TMB had worse prognoses according to the KM curve analysis (Figure 8E).

### Immune landscapes and drug sensitivity in risk scores for CuRLs

**Figure 9:**
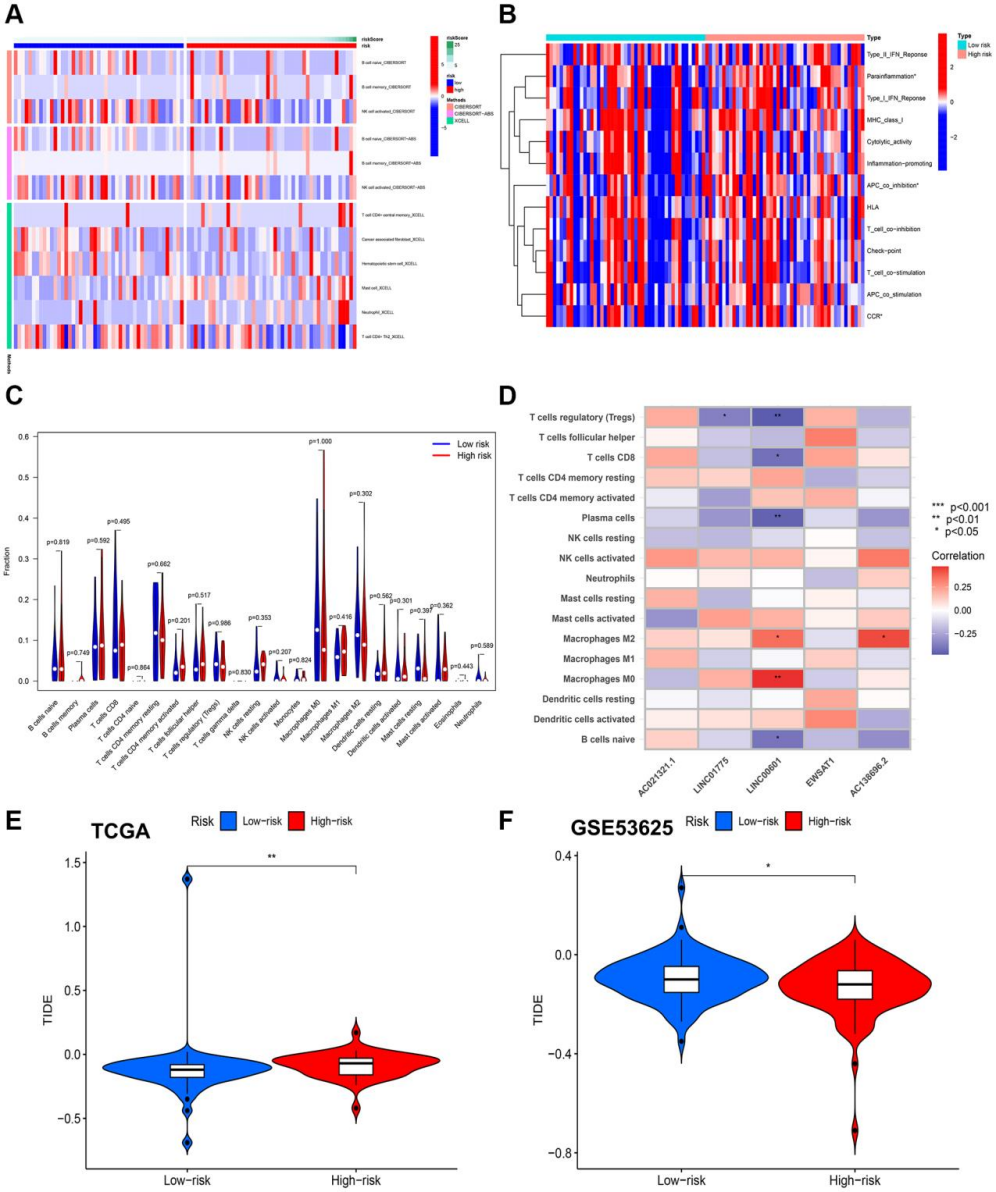
**Results of immunity analysis.** (**A**) Differences in immune infiltration between high- and low-risk groups using TIMER, CIBERSORT, CIBERSORT-ABS, QUANTISEQ, MCPCOUNTER, XCELL and EPIC. (**B**) Differences in expression of common immune checkpoints in the at-risk group. (**C**) Analysis of common immune cell differences in the risk group. (**D**) Analysis of CuRLs and immune cell correlation. (**E**) TIDE scores between the two groups in TCGA group. (**F**) TIDE scores between the two groups in GSE53625 group.

**Figure 10:**
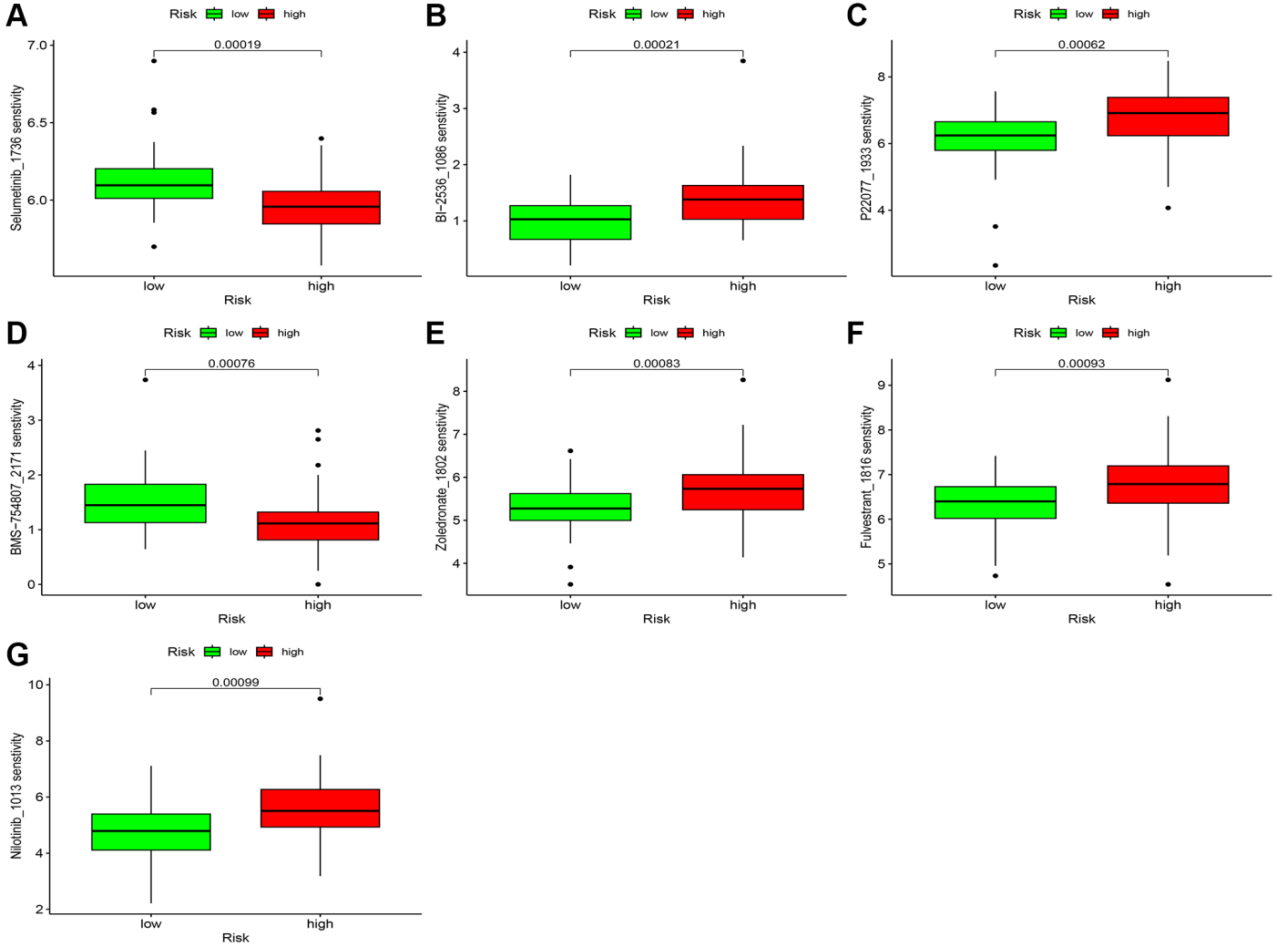
**Chemotherapy sensitivity.** (**A**–**G**) Chemosensitivity difference between two risk groups.

We employed multiple approaches to assess immune infiltration in the high- and low-risk groups (Figure 9A). We investigated the association between the risk score for ESCC and immune-related activities and identified notable variations in the risk score for parainflammation, APC coinhibition, and CCR (Figure 9B, 9C). We also investigated the correlation between the CuRLs and immune pathways and found that LINC00601 was significantly associated with multiple immune pathways (Figure 9D). In fact, TIDE scores were significantly higher in the low-risk group than in the high-risk group in the TCGA cohort (*p* < 0.05), suggesting that patients in the high-risk group were more likely to benefit from immunotherapy. This was validated in the GSE53625 cohort (Figure 9E, 9F). Potential treatment options were predicted by analyzing 198 commonly used chemotherapeutic agents, and we found differences for 66 drugs (*p* < 0.05). Among them, seven drugs (Selumetinib, BI-2536, P22077, BMS-754807, Zoledronate, Fulvestrant, and Nilotinib) had significant associations (*p* < 0.01) (Figure 10A–10G). Sensitivity analysis showed greater sensitivity to Selumetinib and BMS-754807 in the low-risk group than in the high-risk group. These findings suggest that risk assessment can guide personalized drug therapy for ESCC patients (Supplementary Tables 3–5).

### *In vitro* experimental validation of CuRLs as biomarker candidates

To further validate the prognostic potential of the CuRL model, we performed *in vitro* experiments to explore the expression trends of different CuRLs. Our team measured the expression levels of EWSAT1, AC138696.2, AC021321.1, LINC00601, and LINC01775 in KYSE-30, KYSE-410, and KYSE-520 cancer cell lines and in patient cancer tissues and paracancerous tissues using RT-qPCR. The results showed an overall increasing trend in the expression of these lncRNAs (Supplementary Figure 6A–6E), except for LINC01775, which showed no differential expression (Supplementary Figure 7A–7E). The RT-qPCR results are for reference only. Overall, our experimental results support our model.

## DISCUSSION

ESCA is a widespread malignancy that is the ninth worst malignant cancer in the world [1]. Although treatment modalities have evolved in recent years and achieved remarkable results, the prognosis of ESCC patients is still unsatisfactory [23, 24]. The current mainstream prediction model is TNM staging, but it does not meet the actual needs of clinical treatment [25]. Therefore, establishing an accurate prognostic prediction method is urgently needed for the current treatment and diagnosis of ESCC. In this study, we identified CuRLs that play a significant prognostic role and developed a new prognostic signature that can precisely forecast the prognosis of ESCC patients. We found enrichment of B-cell receptor signaling pathways in the high-risk group, specifically the BCR5 pathway. High-risk patients exhibited immunosuppression and high TMB, whereas the two risk groups had varying sensitivity to immunotherapy and chemotherapeutic agents.

This study developed a new prognostic signature using five CuRLs that showed high accuracy in predicting patient outcomes, which is a commonly used approach for constructing prognostic signatures in various cancers, including lung adenocarcinoma and colon cancer [26–28]. A higher risk score was associated with a worse prognosis in ESCC patients, as revealed by our findings. A nomogram was developed to predict patient prognosis by integrating clinical indicators and the risk score, and the model with the risk score exhibited better predictive power than the model without the risk score (C-index: 0.770 vs. 0.686). Furthermore, our prognostic model demonstrated better predictive power than similar models. Among the five identified lncRNAs, EWSAT1 was found to be involved in the development of osteosarcoma and has a role in metastasis, with a significant association with ROCK1, according to Shen et al. [29]. Li et al. reported a significant association between AC021321.1 expression and poor survival as well as immune infiltration in bladder cancer (BC), and it may serve as a prognostic biomarker for BC [30]. LINC00601 was found to be upregulated in hepatocellular carcinoma and promoted the development of the disease through the activation of the MAPK signaling pathway [31]. However, functional studies of the other lncRNAs, namely, LINC01775 and AC138696.2, have not been reported in cancer studies. Finally, RT-qPCR experiments confirmed the significant differential expression of the four CuRLs in normal versus cancer tissues to construct the prognostic model.

In this paper, after performing GSVA with five methods, the high-risk group was observed to have an enrichment of immune-related pathways, including the intestinal immune network for IGA production, B-cell receptor signaling pathway, BCR pathway, antigen activating BCR leading to second messengers, and other immune pathways [32]. The immune response in high-risk patients was enriched in immune-related pathways, indicating a stronger immune response that may contribute to a worse prognosis compared to low-risk patients. Our study also found that lncRNA-based prognostic features were substantially associated with immune cell infiltration, as ssGSEA showed significant activation of immune features (parainflammation, APC coinhibition, and CCR) when the risk score was elevated. These findings suggest that prognostic features may contribute to the discovery of regulatory mechanisms of tumor immunity and provide new insights for future tumor microenvironment (TME) studies. Moreover, TMB is commonly used as an indicative biomarker of immunotherapy for various cancers. Our study revealed that patients with high expression of TP53 and TTN in the high-risk group had a poorer prognosis, which is consistent with the known association between high TMB and worse prognosis [33]. In our study, we also detected specific mutations in the TP53, TTN, and MUC16 genes, with mutation frequencies of 74%, 30%, and 20%, respectively. TP53 is a well-established oncogene that regulates malignancy in ESCC cells. Prior research indicates that mutations in TTN are linked to increased responsiveness of solid tumors to ICIs. Additionally, MUC16 mutations are associated with prognosis and may be related to sites that affect tumor prognosis and progression. The high-risk group in our study demonstrated poorer OS, and mutations identified in this group may impact ESCC development. Hence, the expression of CuRLs with high risk scores could potentially enhance the therapeutic efficacy and prognosis of ESCC patients. In summary, our study highlights the potential of lncRNAs as prognostic markers and elucidates the role of the immune system and TMB in ESCC development and prognosis. The findings of this study could serve as a foundation for future research into the underlying mechanisms of ESCC and the development of more effective treatment strategies.

TIDE is an important tool for predicting the effectiveness of immunotherapy for cancer patients. Previous research has shown that immunotherapy benefits patients with lower TIDE scores [34]. The high-risk group showed a lower TIDE score in our study. Patients in the high-risk group with lower TIDE scores may have an increased potential to benefit from immunotherapy compared to their low-risk counterparts. Currently, chemotherapy and immunotherapy remain the primary treatment options for advanced cancer patients [35]. Due to the specificity of ESCC tumors and the varying effects of different drugs, we conducted drug sensitivity trials and identified five drugs (BI-2536, P22077, Zoledronate, Fulvestrant, and Nilotinib) to which high-risk patients are sensitive. BI-2536 suppresses Plk1 activity at low nanomolar concentrations. In a study by Wu et al., BI-2536 sensitized ESCC cells to DDP by inhibiting DNA damage repair pathways and inducing focal death [36, 37]. While BI-2536 is effective in most ESCC patients, some patients are unable to achieve efficacy due to cancer specificity. We believe that our risk score model can assist in guiding their dosing. P22077 may have anti-inflammatory effects by promoting TRAF48 degradation through K6-linked polyubiquitination [38]. At present, there are no known effects of Zoledronate on ESCC. However, its main mechanism of inhibiting the metastatic progression of ESCC cells involves uptake by the tight junction protein occludin [39]. Nilotinib was found to be a potent inhibitor of ILK by Juan Liu et al. As such, it has the potential to target ILK-mediated signaling pathways and manage ESCC [40].

The strengths of this study are notable and demonstrate its significance in advancing our understanding of cuproptosis-related gene signatures that are predictive of ESCC patient prognosis. This study represents the first attempt to investigate CuRLs as predictors of ESCC patient prognosis. Second, it includes the largest number of cuproptosis-related genes, allowing for a more comprehensive analysis. Third, this study reports a high accuracy in predicting patient prognosis compared to similar models, suggesting the potential clinical utility of CuRLs in ESCC prognosis prediction. Fourth, the study identifies multiple pathways that are simultaneously enriched, providing a more holistic understanding of the mechanisms underlying ESCC. Finally, the use of cell lines to validate CuRLs reduces the interference of external factors and reveals more accurate expression differences. There are limitations to this study: there are no further externally validated data, no siRNA experiments on cancer cell lines, and no animal models to study the effects of cuproptosis. In addition, the function and molecular mechanisms of CuRLs need to be further investigated. Addressing these limitations in future studies will improve the clinical applicability of CuRLs in the prognostic assessment of ESCC.

## CONCLUSIONS

The nomogram model based on the risk score and clinical characteristics was effective in predicting the prognosis of patients. Further mechanistic analysis showed that the high-risk group was enriched in the B-cell receptor signaling pathway and BCR5 pathway and exhibited high TMB expression. In the drug sensitivity analysis, high-risk patients exhibited greater responsiveness to Nilotinib, BI-2536, P22077, Zoledronate, and Fulvestrant. Due to this study’s general shortcomings, the findings need to be confirmed in a large prospective sample and further mechanistic analysis and wet trials.

## Supplementary Materials

Supplementary Figures

Supplementary Tables 1-3 and 5

Supplementary Table 4
