# Supplementary material for: Prognostic value and immune landscapes of cuproptosis-related lncRNAs in esophageal squamous cell carcinoma
Source: Aging (Albany NY). 2023 Oct 6;15(19):10473–500. doi: 10.18632/aging.205089 (PMC10599721; doi:10.18632/aging.205089)
Supplement: Supplementary Figures [file aging-15-205089-s001.pdf]

SUPPLEMENTARY FIGURES

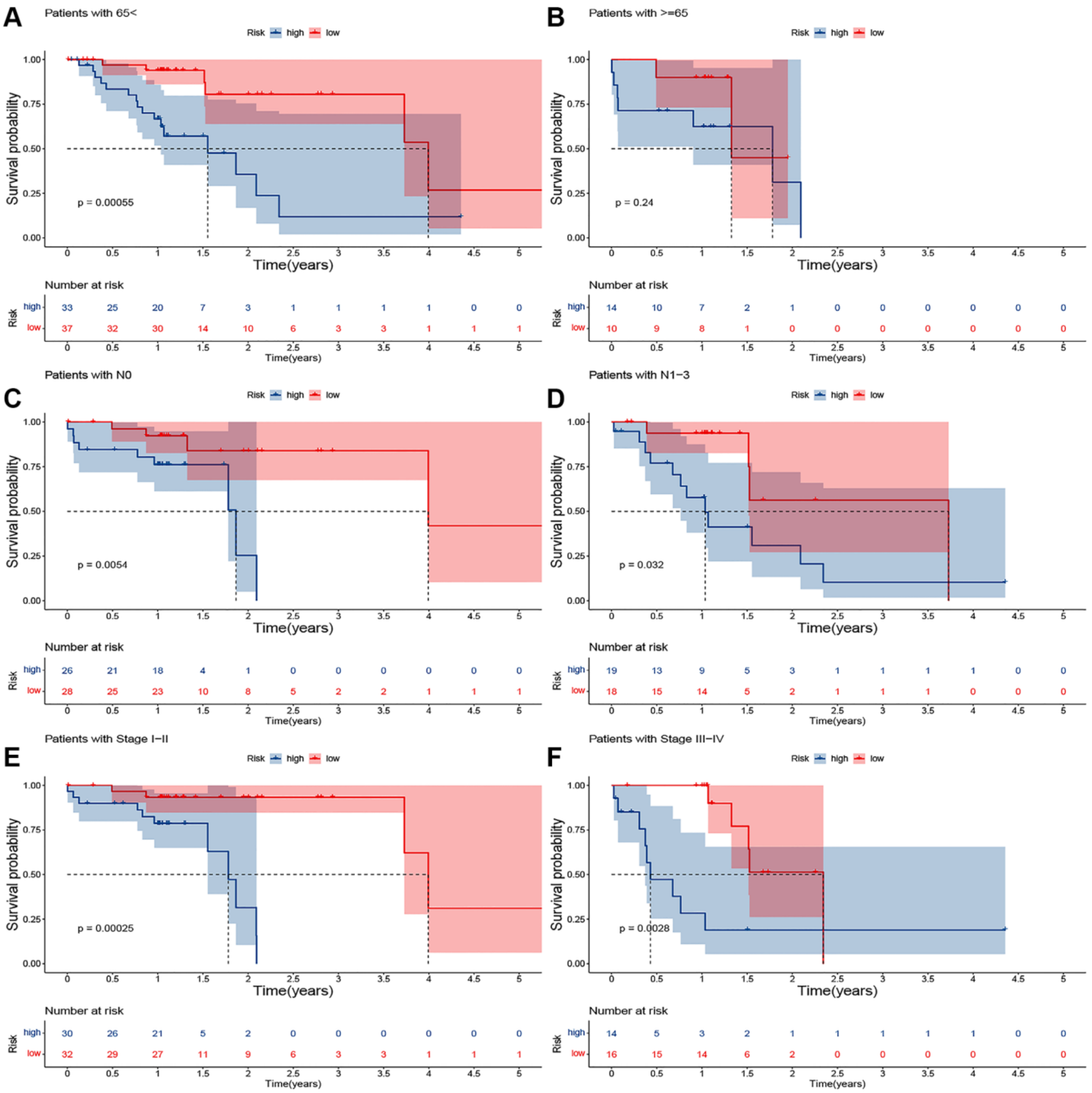

Supplementary Figure 1. Kaplan-Meier survival curves for low- and high-risk populations by different clinical variables. (**A**, **B**) Age; (**C**, **D**) N0 and N1-3; (**E**, **F**) Stage I–II and Stage III–IV.

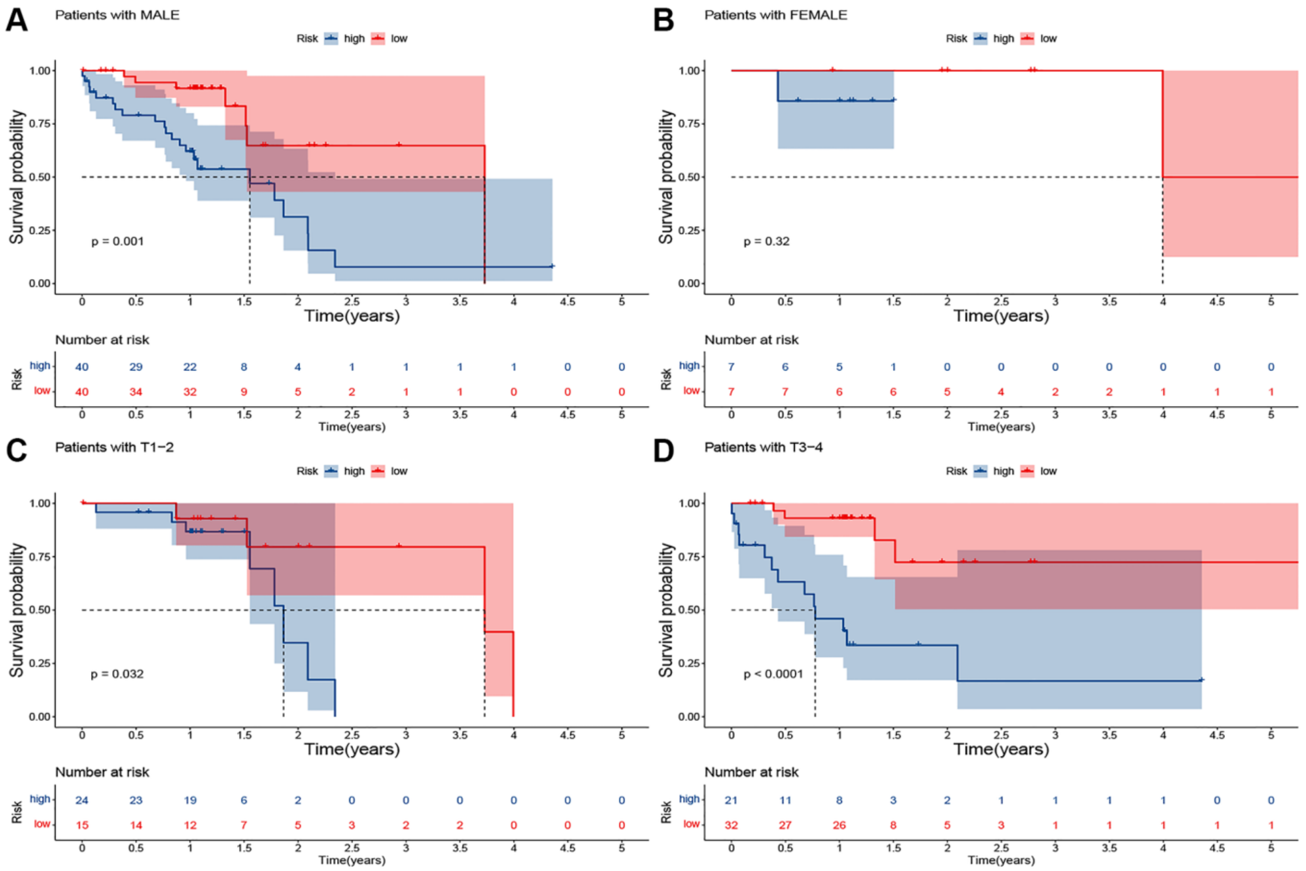

Supplementary Figure 2. Kaplan-Meier survival curves for low- and high-risk populations by different clinical variables. (**A**, **B**) Sex; (**C**, **D**) T1–2 and T3–4.

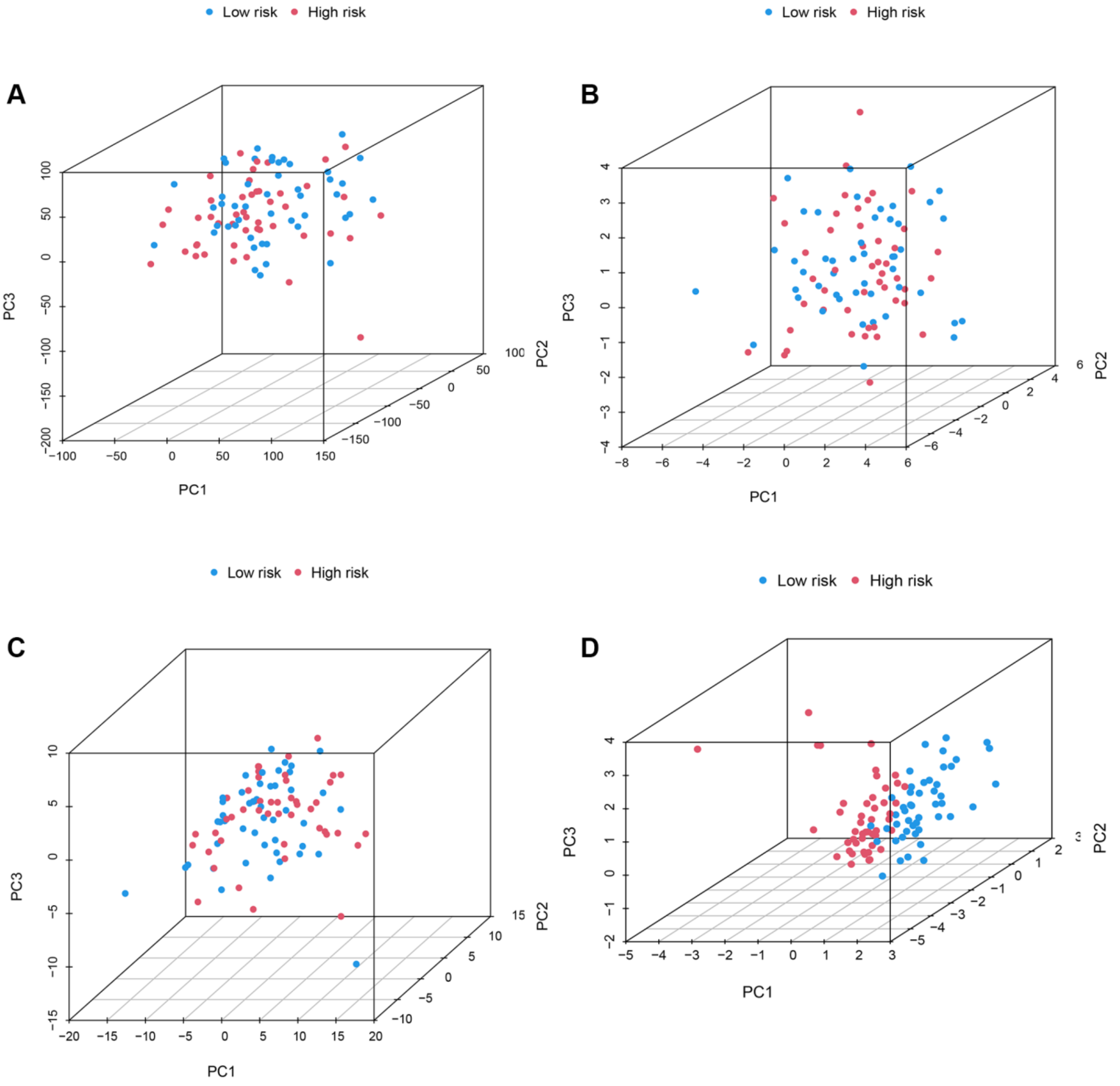

Supplementary Figure 3. PCA analysis of the prognostic signature. (**A**) PCA of all genes; (**B**) PCA of cuproptosis genes; (**C**) PCA of CuRLs; (**D**) PCA of risk LncRNAs.

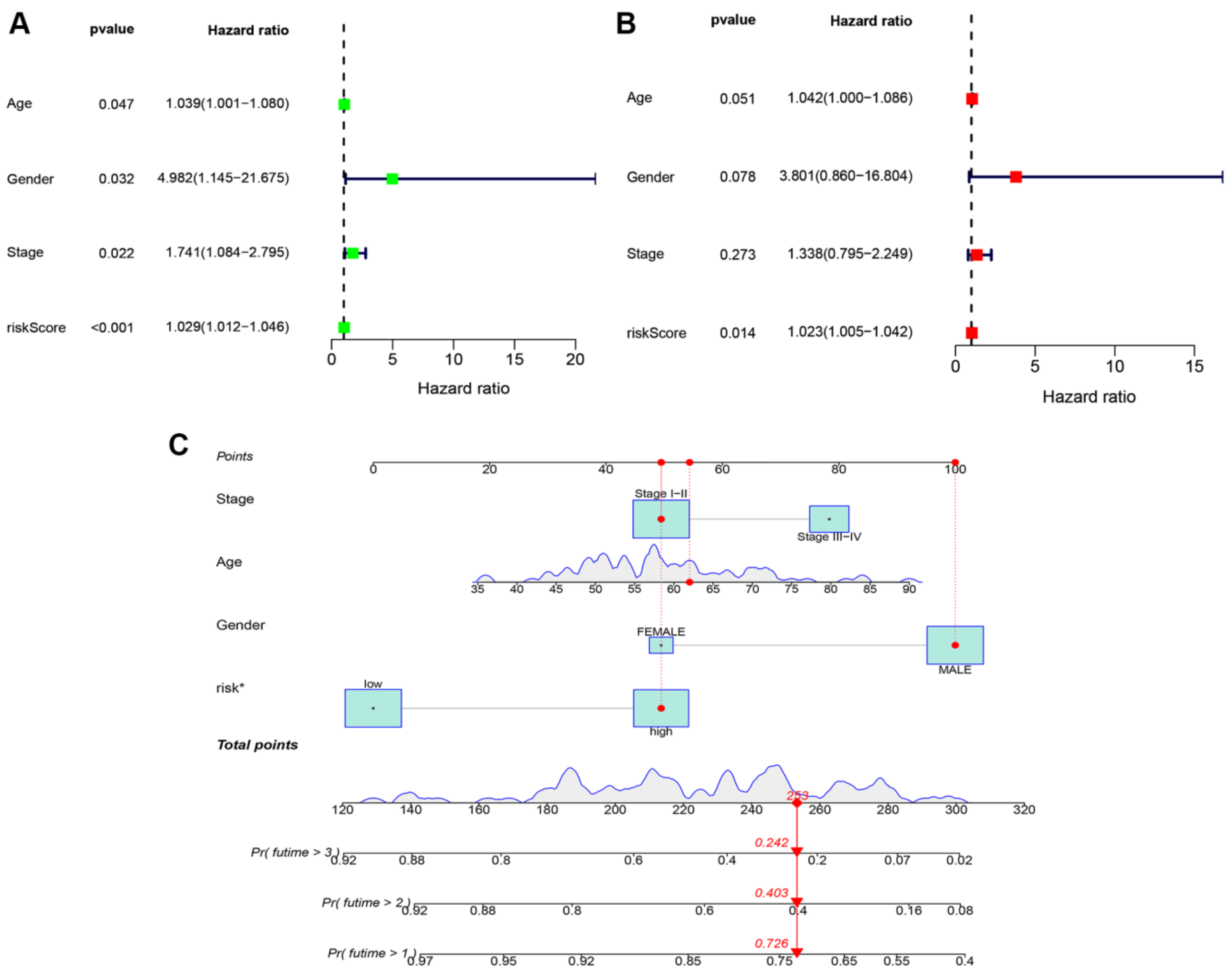

Supplementary Figure 4. Independent prognostic analysis of the prognostic signature. (**A**) Univariate and (**B**) Multivariate Cox regression analysis to examine the value of clinical characteristics and risk score as independent prognostic predictors. Nomograms for predicting 1-, 2-, 3-year overall survival (OS) for a randomly selected patient in test cohort (**C**).

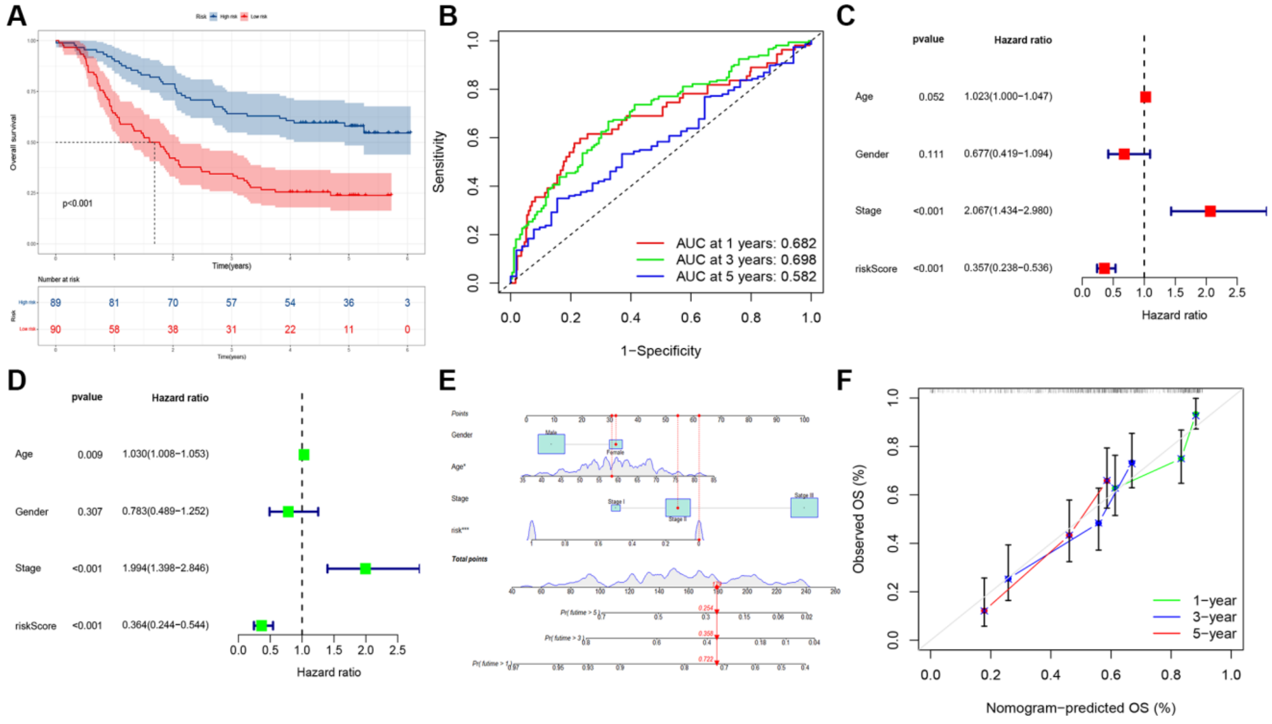

Supplementary Figure 5. Risk model external validation in the GSE53625 group. (**A**) Overall survival Kaplan-Meier survival curves. (**B**) 1-, 3-, and 5-years overall survival area under the ROC curve of the signature. (**C**) Univariate and (**D**) Multivariate Cox regression analysis to examine the value of clinical characteristics and risk score as independent prognostic predictors in GSE53625. (**E**) Nomograms for predicting 1-, 3-, 5-year overall survival (OS) for a randomly selected patient in test cohort. (**F**) Calibration curves for 1, 2, and 3 years of nomogram.

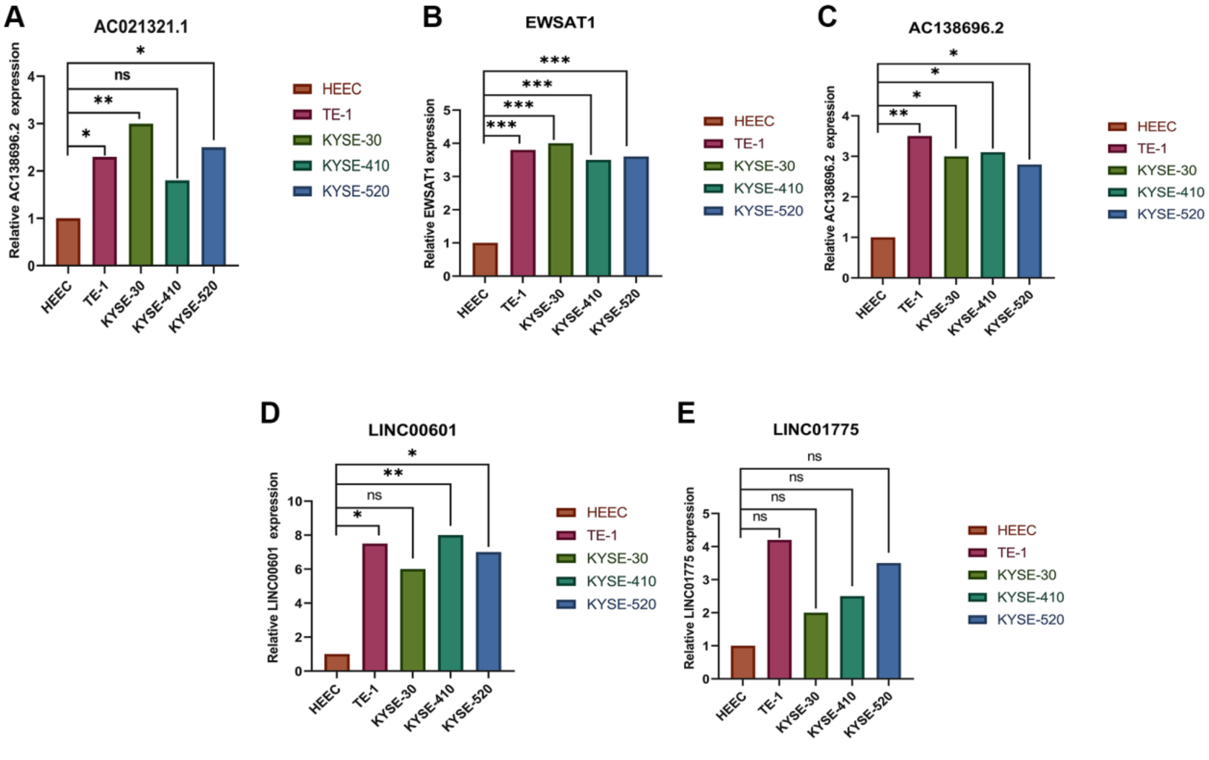

Supplementary Figure 6. Relative mRNA expression of cuproptosis-related long non-coding RNAs in four cell lines (HEEC, TE-1, KYSE-30, KYSE-410 and KYSE-520). (**A**) AC021321.1, (**B**) AC138696.2, (**C**) EWSAT1, (**D**) LINC00601 and (**E**) LINC01775.

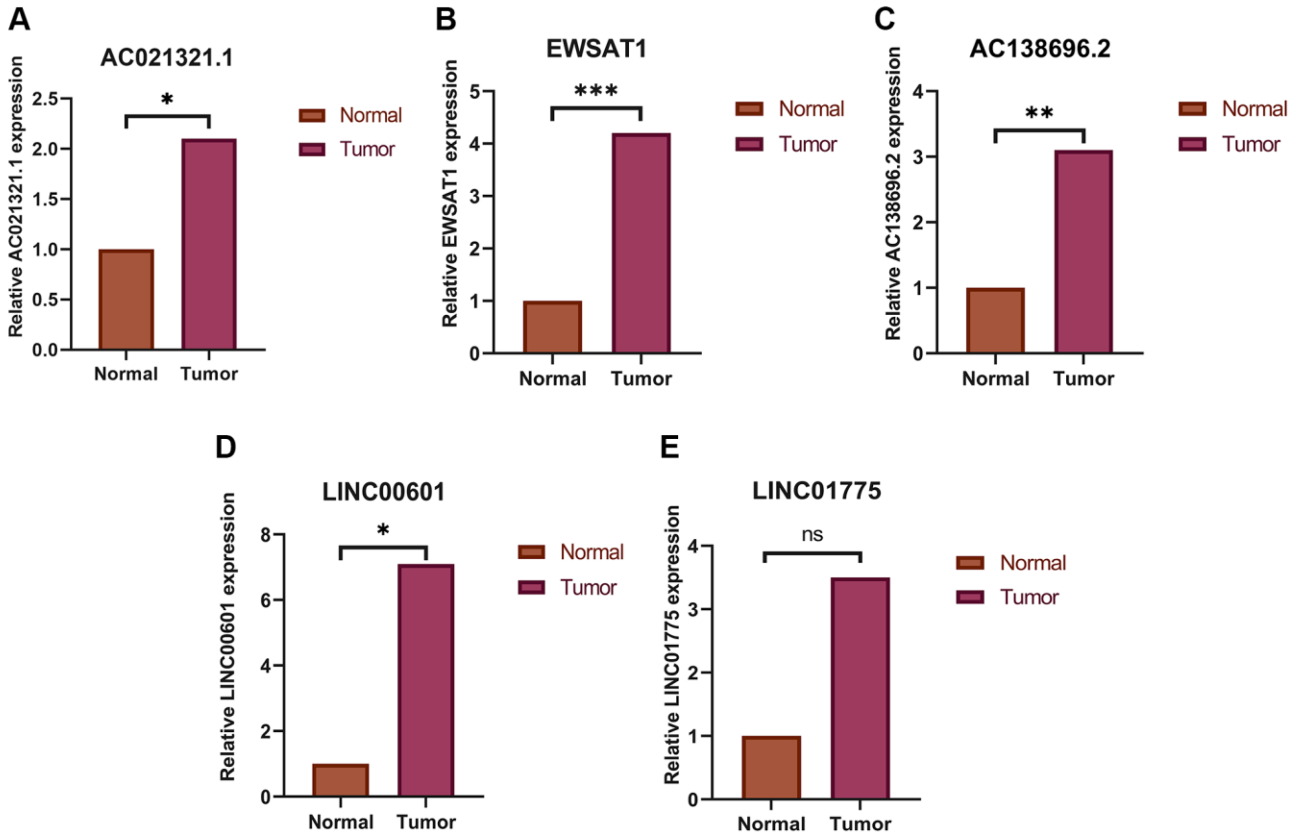

Supplementary Figure 7. Relative mRNA expression of cuproptosis-related long non-coding RNAs in cancer tissue and paracancerous tissue. (**A**) AC021321.1, (**B**) AC138696.2, (**C**) EWSAT1, (**D**) LINC00601 and (**E**) LINC01775.
