# Supplementary material for: Prognostic value and immune landscapes of cuproptosis-related lncRNAs in esophageal squamous cell carcinoma
Source: Aging (Albany NY). 2023 Oct 6;15(19):10473–500. doi: 10.18632/aging.205089 (PMC10599721; doi:10.18632/aging.205089)
Supplement: Supplementary Tables 1-3 and 5 [file aging-15-205089-s002.pdf]

SUPPLEMENTARY TABLES

Supplementary Table 1. Primers for RT-qPCR experiments with 5 cuproptosis-related lncRNAs.

Gene ID	Forward primer	Reverse primer
**AC021321.1**	GGGGCTCATAACAGAGCCAG	AGACAGACTACCGGAGGCAT
**LINC01775**	AGATTCGAGACCCCGGTTTG	AGGTTTGGGTGGGAAATGGG
**LINC00601**	CTTTCTCCCCACAGTCGCTT	AAGTTCCTGATGCGCGAAGA
**EWSAT1**	GTGTCTGGCAAGGAACACTA	GGTGGAGAAGAGGGACAATAAG
**AC138696.2**	GCCTCAGGATGCAACCTTCT	GGATCTCAGCCAAGCAGAGG

Abbreviations: LncRNA: Long non-coding ribonucleic acid; RT-qPCR: Reverse transcription quantitative-polymerase chain reaction.

Supplementary Table 2. 5 CuRLs connected with the over survival of the ESCC patients after multivariate cox analysis in training cohort.

CuRLs	HR	95% CI	*P*-value
**AC021321.1**	0.168	0.029–0.979	0.047
**LINC01775**	0.316	0.115–0.871	0.026
**LINC00601**	2.778	1.295–5.958	0.009
**EWSAT1**	1.756	1.008–2.833	0.021
**AC138696.2**	0.366	0.157–0.851	0.02

Abbreviations: CuRLs: Cuproptosis-related long non-coding RNAs; ESCC: Esophageal squamous cell carcinoma; HR: Hazard ratios; CI: confidence interval.

Supplementary Table 3. IC50 of clinical drug sensitivity for ESCC patients in high and low risk groups.

Drugs	High-risk group		Low-risk group		*P*-value	Sensitive groups
	Media	IQR	Media	IQR		
**ABL signaling**						
Nilotinib	44.46	29.56–76.25	26.68	16.27–41.11	0.00	High
**Apoptosis regulation**						
WEHI-539	42.83	25.52–63.09	30.57	18.46–45.63	0.02	High
Venetoclax	9.87	7.36–11.82	8.23	6.28–10.45	0.02	High
UMI-77	16.05	10.61–24.54	11.97	8.33–18.6	0.03	High
Sepantronium bromide	0.02	0.01–0.04	0.01	0.01–0.02	0.04	High
**Cell cycle**						
AZD7762	1.14	0.73–2.33	0.78	0.55–1.28	0.02	High
Wee1 Inhibitor	9.38	6.09–12.83	6.54	4.19–8.94	0.00	High
RO-3306	18.45	11.62–24.33	12.09	9.92–17.22	0.01	High
BI-2536	1.60	1.04–2.1	1.04	0.59–1.41	0.00	High
MK-1775	2.09	1.31–2.89	1.46	1.07–2.11	0.01	High
MK-8776	24.16	14.91–50.62	20.25	11.46–25.41	0.04	High
**Chromatin histone acetylation**						
Vorinostat	5.06	3.72–6.68	3.69	2.61–4.58	0.00	High
EPZ004777	197.38	132.02–252.74	157.35	115.66–181.22	0.00	High
EPZ5676	293.08	218.71–384.14	225.75	180.71–281.33	0.01	High
PCI-34051	107.68	71.5–170.82	75.64	59.35–116.39	0.03	High

GSK591	107.15	89.97–148.57	82.05	66.16–110.64	0.01	High
**Cytoskeleton**						
BDP-00009066	10.68	8.99–15.34	9.08	6.7–13.05	0.01	High
**Chromatin other**						
PFI3	202.49	177.5–246.39	182.96	156.32–207.78	0.01	High
RVX-208	129.01	103.47–157.32	107.49	87.94–124.78	0.01	High
JQ1	13.43	9.55–19.21	9.58	7.28–14.78	0.03	High
**DNA replication**						
Cyclophosphamide	200.68	143.63–236.85	173.61	124.6–199.26	0.01	High
Gemcitabine	0.67	0.18–3.49	0.24	0.14–0.93	0.01	High
Pyridostatin	31.72	24.46–40.39	24.27	20.07–31.57	0.01	High
**ERK MAPK signaling**						
Trametinib	1.23	0.81–2.41	2.13	1.1–3.96	0.02	Low
Selumetinib	61.14	56.52–65.58	67.39	63.5–72.67	0.00	Low
**EGFR signaling**						
Sapitinib	39.07	17.12–80.46	76.30	33.18–125.05	0.02	Low
**Genome integrity**						
KU-55933	72.18	53.59–87.15	79.96	65.13–110.44	0.04	Low
AZD6738	8.23	5.04–16.28	5.52	3.2–10.15	0.01	High
BIBR-1532	157.32	113.29–228.05	126.51	88.38–158.78	0.01	High
VE821	75.44	48.28–113.77	52.27	26.96–70.33	0.00	High
**Hormone-related**						
Fulvestrant_1200	21.12	16.88–25.65	16.91	14.62–19.72	0.00	High
Fulvestrant_1816	109.56	81.11–145.67	83.50	63.94–105.21	0.00	High
GDC0810	147.82	122.82–195.65	125.93	93.2–152.57	0.02	High
**IGF1R signaling**						
BMS-754807	1.17	0.76–1.5	1.73	1.19–2.55	0.00	Low
**Metabolism**						
Daporinad	0.01	0.01–0.03	0.01	0.01–0.01	0.01	High
**Mitosis**						
Docetaxel	0.01	0.01–0.02	0.01	0.01–0.02	0.03	High
PD173074	75.29	35.57–117.31	43.45	31.07–68.79	0.19	High
Paclitaxel	0.09	0.04–0.16	0.05	0.02–0.08	0.03	High
Tozasertib	21.89	12.85–27.76	16.10	11.48–19.7	0.00	High
Vincristine	0.19	0.11–0.48	0.10	0.06–0.35	0.05	High
**Other**						
Cytarabine	6.77	3.42–17.21	3.66	2.25–7.19	0.01	High
5-Fluorouracil	130.13	92.83–222.36	100.03	37.7–173.73	0.05	High
Pevonedistat	2.46	1.4–4.35	1.32	0.81–2.82	0.01	High
Zoledronate	52.19	36.95–65.81	37.68	30.92–48.21	0.00	High
BPD-00008900	99.34	74.94–135.8	82.71	56.9–100.21	0.01	High
**Other, kinases**						
Sorafenib	16.65	11.66–22.46	14.12	8.79–18.19	0.04	High
JAK1_8709	58.36	39.22–77.12	65.91	57.14–87.95	0.04	Low
GSK2578215A	147.26	121.12–171.83	125.98	103.33–148.47	0.02	High
**p53 pathway**						
MIRA-1	266.91	177.77–375.43	197.15	132.69–249.58	0.01	High
**PI3K/MTOR signaling**						

MK-2206	21.93	16.84–33.07	17.99	14.57–24.66	0.03	High
Dactolisib	0.24	0.16–0.45	0.17	0.08–0.27	0.00	High
Rapamycin	0.15	0.11–0.24	0.10	0.07–0.13	0.00	High
CZC24832	173.99	143.84–199.51	157.30	125.39–180.58	0.05	High
Ipatasertib	40.38	23.59–59.89	27.00	16.73–43.62	0.02	High
AMG-319	145.74	115.24–181.25	116.73	99.55–139.35	0.01	High
Uprosertib_2106	20.02	11.83–34.05	14.41	7.87–18.5	0.02	High
LJI308	184.82	151.1–218.79	151.09	130.03–176.65	0.03	High
AT13148	51.37	25.33–75.28	29.71	19.8–49.09	0.03	High
**Protein stability and degradation**						
P22077	119.56	74.37–166.27	74.76	54.59–99.64	0.00	High
**RTK signaling**						
Axitinib	28.54	28.54–28.54	28.54	28.54–28.54	0.02	High
Crizotinib	27.50	20.07–42.43	19.93	15.59–29.16	0.01	High
AZD4547	19.74	12.64–33.19	14.67	10.14–21.28	0.01	High
Cediranib	9.68	6.84–12.02	7.63	6.23–10.33	0.05	High
**Unclassified**						
Gallibiscoquinazole	16.42	12.5–20.99	11.91	9.77–16.2	0.00	High
**WNT signaling**						
SB216763	185.20	162.54–207.12	200.15	180.43–224.37	0.04	Low
MN-64	125.20	92.03–158.05	93.06	73.9–133.96	0.02	High

Abbreviations: ESCC: Esophageal squamous cell carcinoma; IC50: half maximal inhibitory concentration; IQR: Interquartile range.

Supplementary Table 5. Summary of anti-tumor drug categories.

Category	A: Number of high-risk sensitive	B: Number of significant differences	C: Total quantity of drugs	B/C	A/B
ABL signaling	1	1	1	100	100
Apoptosis regulation	4	4	12	33.33	25
Cell cycle	6	6	12	50	100
Chromatin histone acetylation	5	5	8	62.5	100
Chromatin other	3	3	7	42.86	100
Cytoskeleton	1	1	3	33.33	100
DNA replication	3	3	15	20	100
EGFR signaling	0	1	7	14.29	NULL
ERK MAPK signaling	0	2	12	16.67	NULL
Genome integrity	3	4	11	36.36	75
Hormone-related	3	3	4	75	100
IGF1R signaling	0	1	6	16.67	NULL
JNK and p38 signaling	0	0	1	0	NULL
Metabolism	1	1	4	25	100
Mitosis	5	5	9	55.56	100
Other	5	5	15	33.33	100
Other, kinases	2	3	14	21.43	66.67
p53 pathways	1	1	3	33.33	100
PI3K/MTOR signaling	9	9	22	40.91	100

Protein stability and degradation	1	1	5	20	100
RTK signaling	4	4	11	36.36	100
Unclassified	1	1	6	16.67	100
WNT signaling	1	2	9	22.22	50
