# Supplementary material for: Prognostic value and immune landscapes of cuproptosis-related lncRNAs in esophageal squamous cell carcinoma
Source: Aging (Albany NY). 2023 Oct 6;15(19):10473–500. doi: 10.18632/aging.205089 (PMC10599721; doi:10.18632/aging.205089)
Supplement: Supplementary Table 4 [file aging-15-205089-s003.docx]

**Supplementary Table 4. IC50 of clinical drug sensitivity for ESCC patients in high and low risk groups with no difference.**

| **Drugs** | **High-risk group** | |  | **Low-risk group** | | **P-value** |
| --- | --- | --- | --- | --- | --- | --- |
|  | **Media** | **IQR** |  | **Media** | **IQR** |  |
| **Apoptosis regulation** | |  |  |  |  |  |
| MIM1 | 47.57 | 42.08-70.65 |  | 48.19 | 31.57-62.68 | 0.32 |
| AZD5991 | 67.53 | 36.81-193.87 |  | 83.62 | 33.47-135.1 | 0.73 |
| Obatoclax Mesylate | 4.34 | 2.65-5.89 |  | 3.98 | 2.66-6.25 | 0.63 |
| Sabutoclax | 0.65 | 0.41-1.11 |  | 0.68 | 0.36-1.07 | 0.87 |
| AZD5582 | 9.52 | 4.01-25.1 |  | 7.82 | 3.19-20.32 | 0.30 |
| Navitoclax | 7.68 | 5.08-11.65 |  | 5.93 | 3.05-10.67 | 0.06 |
| LCL161 | 150.47 | 119.51-207.28 |  | 127.28 | 94.63-174.24 | 0.09 |
| ABT737 | 11.56 | 5.07-18.05 |  | 6.35 | 4.35-12.85 | 0.07 |
| **Cell cycle** |  |  |  |  |  |  |
| Ribociclib | 47.16 | 43.97-51.07 |  | 48.70 | 46.52-51.04 | 0.07 |
| AZD5438 | 8.22 | 6.39-14.29 |  | 7.80 | 5.78-13.26 | 0.63 |
| CDK9_5038 | 0.09 | 0.06-0.17 |  | 0.08 | 0.05-0.15 | 0.61 |
| Palbociclib | 45.09 | 30.25-64.31 |  | 36.15 | 24.4-47.82 | 0.07 |
| Dinaciclib | 0.06 | 0.03-0.09 |  | 0.05 | 0.04-0.1 | 0.93 |
| CDK9_5576 | 0.55 | 0.38-1.12 |  | 0.66 | 0.43-0.94 | 0.69 |
| **Chromatin histone acetylation** | | |  |  |  |  |
| Entinostat | 7.94 | 3.88-19.13 |  | 10.39 | 6.84-15.06 | 0.17 |
| GSK343 | 18.22 | 15.31-21.33 |  | 16.51 | 13.51-18.93 | 0.12 |
| OF-1 | 57.98 | 45.91-89.11 |  | 62.50 | 47.57-81.08 | 0.95 |
| **Chromatin other** | |  |  |  | - |  |
| OTX015 | 12.48 | 8.57-18.47 |  | 10.47 | 7.46-17.36 | 0.52 |
| I-BET-762 | 30.77 | 21.86-41.26 |  | 24.55 | 18.92-36.26 | 0.33 |
| AZD5153 | 6.08 | 3.85-7.83 |  | 4.64 | 3.73-7.47 | 0.27 |
| I-BRD9 | 87.76 | 68.51-106.01 |  | 72.69 | 54.33-91.28 | 0.05 |
| **Cytoskeleton** | |  |  |  |  |  |
| GSK269962A | 20.17 | 14.67-24.18 |  | 18.47 | 15.91-20.88 | 0.36 |
| PAK_5339 | 11.51 | 8.96-13.76 |  | 11.02 | 8.56-13.7 | 0.43 |
| **DNA replication** | |  |  |  |  |  |
| Cisplatin | 26.19 | 18.18-51.11 |  | 20.20 | 10.03-44.12 | 0.25 |
| Fludarabine | 141.92 | 95.92-248.49 |  | 165.89 | 117.51-192.33 | 0.98 |
| Oxaliplatin | 159.50 | 124.89-221.96 |  | 150.60 | 99.44-215.27 | 0.45 |
| Nelarabine | 455.43 | 335.8-608.76 |  | 365.05 | 263.5-531.67 | 0.08 |
| Irinotecan | 14.43 | 8.61-25.88 |  | 11.63 | 6.01-25.23 | 0.32 |
| Epirubicin | 0.38 | 0.28-0.7 |  | 0.36 | 0.19-0.54 | 0.18 |
| Topotecan | 0.97 | 0.47-2.06 |  | 1.36 | 0.51-2.33 | 0.58 |
| Temozolomide | 434.01 | 309.08-545.26 |  | 392.69 | 318.94-461.05 | 0.38 |
| Leflunomide | 156.69 | 118.02-208.5 |  | 152.27 | 115.6-178.74 | 0.60 |
| Teniposide | 1.89 | 1.05-2.92 |  | 1.54 | 0.54-3.26 | 0.33 |
| Mitoxantrone | 1.66 | 1.1-3.01 |  | 1.79 | 1.19-3.18 | 0.67 |
| **EGFR signaling** | |  |  |  |  |  |
| Gefitinib | 22.28 | 9.91-38.8 |  | 32.49 | 19.18-50.33 | 0.10 |
| Afatinib | 5.73 | 2.74-10.49 |  | 7.74 | 5.45-10.67 | 0.12 |
| Erlotinib | 12.72 | 5.04-20 |  | 16.31 | 9.93-28.02 | 0.07 |
| Lapatinib | 16.23 | 8.53-32.73 |  | 21.67 | 16.24-33.67 | 0.16 |
| AZD3759 | 13.68 | 6.25-22.88 |  | 17.85 | 12.53-24.92 | 0.10 |
| Osimertinib | 5.61 | 2.64-8.63 |  | 6.34 | 4.3-9.79 | 0.25 |
| **ERK MAPK signaling** | | |  |  |  |  |
| ERK_2440 | 10.15 | 6.8-23.57 |  | 15.65 | 8.51-27.71 | 0.39 |
| ERK_6604 | 28.54 | 18.8-41.42 |  | 29.77 | 22.33-54.43 | 0.20 |
| Ulixertinib | 16.35 | 9.61-23.43 |  | 16.67 | 12.02-25.6 | 0.28 |
| Ulixertinib_2047 | 9.44 | 6.95-13.68 |  | 8.16 | 6.7-11.18 | 0.28 |
| VX-11e | 16.52 | 12.46-23.86 |  | 16.46 | 10.9-26.76 | 0.95 |
| SCH772984 | 14.42 | 7.55-18.85 |  | 14.74 | 6.98-27.98 | 0.37 |
| PD0325901 | 1.42 | 0.97-2.29 |  | 2.03 | 1.21-2.76 | 0.06 |
| KRAS (G12C) Inhibitor-12 | 93.96 | 48.2-170.9 |  | 66.08 | 49.42-99.28 | 0.13 |
| Dabrafenib | 114.61 | 73.8-150.69 |  | 102.56 | 71.68-124.26 | 0.20 |
| PLX-4720 | 94.35 | 68.44-138.8 |  | 76.26 | 57.67-96.53 | 0.05 |
| **Genome integrity** | |  |  |  |  |  |
| NU7441 | 15.81 | 14.94-16.4 |  | 15.41 | 14.9-16.14 | 0.78 |
| Talazoparib | 27.57 | 15.97-44.81 |  | 18.71 | 11.21-40.64 | 0.16 |
| Telomerase Inhibitor IX | 1.65 | 1.32-2.6 |  | 1.40 | 1.01-1.99 | 0.05 |
| Niraparib | 74.44 | 43.47-107.92 |  | 83.18 | 60.18-108.76 | 0.45 |
| Mirin | 114.91 | 68.34-165.9 |  | 123.50 | 79.58-161.87 | 0.56 |
| Olaparib | 76.12 | 60.35-118.85 |  | 72.50 | 51.96-98.9 | 0.28 |
| VE-822 | 25.61 | 16.92-42.74 |  | 29.97 | 19.51-44.21 | 0.60 |
| **Hormone-related** | |  |  |  |  |  |
| Tamoxifen | 38.28 | 30.65-44.17 |  | 33.21 | 27.05-40 | 0.07 |
| **IGF1R signaling** | |  |  |  |  |  |
| IGF1R_3801 | 5.59 | 3.91-8.11 |  | 4.76 | 3.11-8.55 | 0.62 |
| BMS-536924 | 8.31 | 6.31-10.76 |  | 9.01 | 5.98-11.42 | 0.79 |
| GSK1904529A | 79.83 | 62.34-119.56 |  | 71.40 | 50.05-91.07 | 0.06 |
| NVP-ADW742 | 16.06 | 11.98-26.42 |  | 14.53 | 10.13-21.62 | 0.18 |
| Linsitinib | 40.16 | 32.43-57.11 |  | 46.58 | 34.31-58.18 | 0.76 |
| **JNK and p38 signaling** | | |  |  |  |  |
| Doramapimod | 92.63 | 78.67-116.07 |  | 97.08 | 78.83-115.21 | 0.89 |
| **Metabolism** | |  |  |  |  |  |
| AGI-5198 | 102.18 | 85.31-132.92 |  | 104.06 | 89.54-120.91 | 0.96 |
| AGI-6780 | 66.05 | 49.64-88.35 |  | 62.68 | 46.88-71.76 | 0.21 |
| GSK2606414 | 43.53 | 33.48-62.1 |  | 38.32 | 30.55-51.93 | 0.26 |
| **Mitosis** |  |  |  |  |  |  |
| Docetaxel_1819 | 0.11 | 0.06-0.28 |  | 0.05 | 0.03-0.32 | 0.11 |
| ZM447439 | 18.17 | 14.63-26.3 |  | 17.89 | 15.27-23.26 | 0.74 |
| Vinblastine | 0.03 | 0.02-0.05 |  | 0.02 | 0.01-0.03 | 0.09 |
| Alisertib | 5.07 | 2.83-12.72 |  | 8.73 | 5.05-16.16 | 0.12 |
| **Other** |  |  |  |  |  |  |
| LY2109761 | 186.93 | 130.3-261.3 |  | 163.27 | 114.46-207.73 | 0.16 |
| VSP34_8731 | 10.89 | 5.31-19.12 |  | 12.88 | 6.89-17.09 | 0.34 |
| Picolinici-acid | 164.77 | 129.1-224.06 |  | 170.39 | 143.39-204.61 | 0.96 |
| TAF1_5496 | 40.78 | 25.7-74.91 |  | 52.62 | 37.24-75.51 | 0.37 |
| IAP_5620 | 198.06 | 126.07-315.93 |  | 158.89 | 96.56-242.71 | 0.16 |
| YK-4-279 | 11.05 | 6.63-18.37 |  | 6.75 | 4.6-13.62 | 0.07 |
| BMS-345541 | 26.98 | 18.09-50.65 |  | 26.58 | 18.89-51.19 | 0.71 |
| Dactinomycin | 0.10 | 0.07-0.13 |  | 0.09 | 0.05-0.12 | 0.40 |
| Dactinomycin_1911 | 0.01 | 0.01-0.02 |  | 0.01 | 0.01-0.01 | 0.49 |
| Eg5_9814 | 0.05 | 0.03-0.07 |  | 0.04 | 0.03-0.07 | 0.28 |
| **Other, kinases** | |  |  |  |  |  |
| AZD1208 | 212.11 | 157.77-265.78 |  | 189.11 | 157.48-234.39 | 0.25 |
| JAK_8517 | 23.04 | 12.14-31.26 |  | 19.35 | 11.92-25.56 | 0.28 |
| Dasatinib | 6.18 | 2.45-11.18 |  | 4.82 | 2.37-9 | 0.64 |
| AZ960 | 8.57 | 4.46-13.81 |  | 6.90 | 4.5-10.95 | 0.53 |
| Entospletinib | 45.37 | 28.68-60.87 |  | 37.54 | 28.51-52.12 | 0.18 |
| Ruxolitinib | 136.08 | 90.35-172.77 |  | 122.67 | 101.61-152.72 | 0.67 |
| ULK1_4989 | 10.10 | 6.34-14.72 |  | 10.11 | 7.15-13.29 | 0.84 |
| AZD5363 | 22.09 | 12.69-32.65 |  | 16.56 | 11.62-25.61 | 0.19 |
| IRAK4_4710 | 146.17 | 113.91-171.86 |  | 135.17 | 118.18-158.96 | 0.53 |
| PRT062607 | 25.66 | 20.94-30.99 |  | 26.60 | 21.3-32.34 | 0.99 |
| Ibrutinib | 80.95 | 43.97-155.44 |  | 107.16 | 59.54-144.63 | 0.34 |
| **p53 pathway** | |  |  |  |  |  |
| Nutlin-3a (-) | 118.51 | 55.56-218.88 |  | 95.58 | 54.1-150.36 | 0.30 |
| PRIMA-1MET | 83.38 | 42.17-167.95 |  | 97.42 | 51.74-195.03 | 0.34 |
| **PI3K/MTOR signaling** | | |  |  |  |  |
| AZD8055 | 0.86 | 0.77-0.91 |  | 0.85 | 0.77-0.92 | 0.82 |
| Afuresertib | 11.54 | 9.05-20.35 |  | 11.24 | 8.69-17.02 | 0.43 |
| PF-4708671 | 50.88 | 40.18-58.34 |  | 50.63 | 44.29-58.51 | 0.40 |
| AZD6482 | 25.21 | 20.94-29.87 |  | 26.69 | 22.27-30.91 | 0.22 |
| GNE-317 | 1.73 | 1.36-2.36 |  | 1.65 | 1.26-2.01 | 0.35 |
| Buparlisib | 2.58 | 2.17-3.26 |  | 2.47 | 1.97-3.23 | 0.52 |
| Pictilisib | 4.31 | 2.84-6.48 |  | 4.05 | 2.74-5.68 | 0.39 |
| AZD2014 | 7.60 | 5.34-11.69 |  | 7.90 | 5.26-11.4 | 0.90 |
| Uprosertib_1553 | 16.79 | 12.42-26.98 |  | 19.33 | 12.1-31.46 | 0.68 |
| Alpelisib | 37.19 | 23.8-54.92 |  | 38.02 | 22.74-49.71 | 0.63 |
| Taselisib | 6.75 | 4.14-13.47 |  | 8.72 | 5.09-14.13 | 0.45 |
| OSI-027 | 108.33 | 81.29-165.82 |  | 131.41 | 92.97-181.63 | 0.37 |
| AZD8186 | 24.69 | 16.7-35.2 |  | 30.03 | 19.83-39.32 | 0.17 |
| **Protein stability and degradation** | | |  |  |  |  |
| ML323 | 88.62 | 67.17-122.88 |  | 90.81 | 59.53-111.39 | 0.28 |
| Luminespib | 0.09 | 0.06-0.2 |  | 0.09 | 0.04-0.19 | 0.49 |
| Bortezomib | 0.01 | 0.01-0.01 |  | 0.01 | 0.01-0.01 | 0.14 |
| MG-132 | 0.19 | 0.17-0.24 |  | 0.18 | 0.15-0.24 | 0.33 |
| **RTK signaling** | |  |  |  |  |  |
| AZD1332 | 51.77 | 33.85-77.1 |  | 42.74 | 28.59-62.97 | 0.14 |
| Savolitinib | 13.55 | 10.83-17.62 |  | 12.69 | 9.25-17.31 | 0.41 |
| Foretinib | 2.89 | 2.29-3.78 |  | 2.37 | 1.62-3.56 | 0.08 |
| SB505124 | 9.11 | 6.03-15.57 |  | 9.61 | 7.02-12.96 | 0.95 |
| Staurosporine | 0.04 | 0.03-0.08 |  | 0.04 | 0.03-0.08 | 0.89 |
| **Unclassified** | |  |  |  |  |  |
| Sinularin | 35.68 | 27.72-48.1 |  | 37.12 | 27.42-45.24 | 0.68 |
| Acetalax | 111.68 | 67.74-187.02 |  | 129.71 | 72.69-234.32 | 0.58 |
| Dihydrorotenone | 2.50 | 1.4-4.27 |  | 2.74 | 1.98-3.58 | 0.98 |
| Elephantin | 23.04 | 14.8-58.46 |  | 27.05 | 15.79-51.63 | 0.91 |
| Podophyllotoxin bromide | 0.58 | 0.39-0.89 |  | 0.41 | 0.3-0.73 | 0.05 |
| **WNT signaling** | |  |  |  |  |  |
| Carmustine | 458.02 | 381.39-641.86 |  | 463.25 | 340.12-536.47 | 0.23 |
| WIKI4 | 39.21 | 34.36-45.83 |  | 42.84 | 36.92-47.19 | 0.21 |
| AZ6102 | 11.88 | 9.02-17.54 |  | 11.10 | 8.96-13.2 | 0.16 |
| Wnt-C59 | 71.76 | 53.39-95.46 |  | 67.93 | 52.83-83.86 | 0.39 |
| LGK974 | 51.54 | 35.6-78.89 |  | 56.22 | 46.22-85.18 | 0.32 |
| IWP-2 | 15.30 | 11.86-21.09 |  | 16.30 | 12.7-20.57 | 0.95 |
| XAV939 | 76.85 | 62.99-103.1 |  | 79.57 | 66-95.34 | 0.62 |

**Abbreviation:** ESCC: esophageal squamous cell carcinoma; IC50: Half maximal inhibitory concentration; IQR: Interquartile range.
